# TMCO1-mediated Ca^2+^ leak underlies osteoblast functions via CaMKII signaling

**DOI:** 10.1038/s41467-019-09653-5

**Published:** 2019-04-08

**Authors:** Jianwei Li, Caizhi Liu, Yuheng Li, Qiaoxia Zheng, Youjia Xu, Beibei Liu, Weijia Sun, Yuan Li, Shuhui Ji, Mingwei Liu, Jing Zhang, Dingsheng Zhao, Ruikai Du, Zizhong Liu, Guohui Zhong, Cuiwei Sun, Yanqing Wang, Jinping Song, Shu Zhang, Jun Qin, Shukuan Ling, Xianhua Wang, Yingxian Li

**Affiliations:** 1 State Key Laboratory of Space Medicine Fundamentals and Application, China Astronaut Research and Training Center, Beijing, 100094 China; 2 The Key Laboratory of Aerospace Medicine, Ministry of Education, The Fourth Military Medical University, 710032 Xi’an, China; 3 State Key Laboratory of Membrane Biology, Beijing Key Laboratory of Cardiometabolic Molecular Medicine, Peking-Tsinghua Center for Life Sciences, Institute of Molecular Medicine, Peking University, Beijing, 100871 China; 4 The Second Affiliated Hospital of Soochow University, Suzhou, 215123 China; 5 State Key Laboratory of Proteomics, Beijing Proteome Research Center, Beijing Institute of Radiation Medicine, Beijing, 100850 China; 6 School of Life Sciences, Tsinghua University, Beijing, 100084 China

## Abstract

Transmembrane and coiled-coil domains 1 (TMCO1) is a recently identified Ca^2+^ leak channel in the endoplasmic reticulum. TMCO1 dysfunction in humans is associated with dysmorphism, mental retardation, glaucoma and the occurrence of cancer. Here we show an essential role of TMCO1 in osteogenesis mediated by local Ca^2+^/CaMKII signaling in osteoblasts. TMCO1 levels were significantly decreased in bone from both osteoporosis patients and bone-loss mouse models. *Tmco1*^−/−^ mice exhibited loss of bone mass and altered microarchitecture characteristic of osteoporosis. In the absence of TMCO1, decreased HDAC4 phosphorylation resulted in nuclear enrichment of HADC4, which leads to deacetylation and degradation of RUNX2, the master regulator of osteogenesis. We further demonstrate that TMCO1-mediated Ca^2+^ leak provides local Ca^2+^ signals to activate the CaMKII-HDAC4-RUNX2 signaling axis. The establishment of TMCO1 as a pivotal player in osteogenesis uncovers a novel potential therapeutic target for ameliorating osteoporosis.

## Abstract (web summary)

TMCO1 is a recently described endoplasmic reticular Ca^2+^ channel. Here, the authors show it is important for osteoblast function and bone formation in mice, and identify a novel pathway linking local increases in Ca^2+^ at the ER surface with the posttranslational modification of RUNX2.

## Introduction

Osteoporosis is a disease that develops with age and is characterized by systemic impairment of bone mass and microarchitecture. Bone metabolism disorders, with reductions in bone formation by osteoblasts, play an important role in this process. However, the molecular mechanisms that mediate impaired bone formation are poorly understood. Many transcription factors are known to be involved in the regulation of osteoblast function in response to different cellular stimuli, such as RUNX2, ATF4, Osterix, and AP1^1–5^. These transcription factors are regulated by a range of developmental signals and play important roles during various stages of cellular development of osteoblast lineages. Among them, RUNX2 is essential for differentiation of mesenchymal cells into osteoblasts and inhibition of osteoblast differentiation into adipocytes and chondrocytes. As the master regulator of osteoblast differentiation, RUNX2 is tightly regulated at both the transcriptional and post-translational levels during the bone formation processes^6^.

Ca^2+^ signaling is essential for bone remodeling. Release of calcium from the endoplasmic reticulum (ER) to trigger Ca^2+^ signaling plays a significant role in osteoblast proliferation and differentiation. The effects of Ca^2+^ are mediated by the Ca^2+^ binding protein calmodulin (CaM). Calmodulin-dependent kinase II (CaMKII) is the major target of Ca^2+^/CaM. After binding to Ca^2+^/CaM, CaMKII is phosphorylated and activates the CREB/ATF and ERK signaling pathways, which induce changes in osteoblast functions^7^. Calcineurin (Cn) is another protein complex that responds to Ca^2+^ signaling. Upon binding to Ca^2+^/CaM, Cn directly binds to and dephosphorylates the NFAT transcription factors that regulate osteoblast differentiation^8,9^. The mechanisms by which changes in Ca^2+^ signaling regulate osteoblastic-specific transcription factors remain unknown.

Release of Ca^2+^ from intracellular stores is facilitated by a series of ion channels in the ER. However, little is known about how intracellular Ca^2+^ signaling is regulated by these channels during osteoblast differentiation. Transmembrane and coiled-coil domain 1 (TMCO1) have been reported to be a highly conserved, transmembrane-spanning protein located in the ER^10^. Loss of TMCO1 function has been identified as the pathogenic cause of an autosomal-recessive syndrome characterized by craniofacial dysmorphism, skeletal anomalies, and intellectual disability^11–13^. Further research has demonstrated that TMCO1 acts as a Ca^2+^ channel, preventing intracellular Ca^2+^ stores from overfilling and maintaining calcium homeostasis in the ER^10^. Ca^2+^ homeostasis in the ER is important for the intracellular Ca^2+^ signaling and is involved in the regulation of a variety of cellular processes, such as proliferation, differentiation, and programmed cell death, in bone cells^14,15^. Disarrangement of ER Ca^2+^ homeostasis is related to many severe bone diseases^16–19^. The role of TMCO1 in bone formation and remodeling remains unclear.

In this study, we found that TMCO1 levels were significantly decreased in bone specimens from both osteoporosis patients and osteoporotic mice. TMCO1 deficiency inhibited osteoblast differentiation and bone formation in vivo and in vitro. RUNX2, one of the primary transcription factors required for osteoblast function^1^, was found to mediate the effects of TMCO1 on osteoblast function. We further demonstrated that loss of TMCO1 in osteoblasts disrupted ER Ca^2+^ homeostasis and promoted CaMKII-HDAC4 axis-mediated RUNX2 degradation in a local Ca^2+^ signaling-dependent manner. These results define the critical functions of TMCO1 in osteoblasts and highlight the importance of Ca^2+^ signaling in RUNX2 protein stability regulation, which suggests a key role for TMCO1 in the pathophysiological process that leads to reduced bone formation in osteoporosis.

## Results

### TMCO1 deficiency inhibits bone formation in vivo

**Fig. 1:**
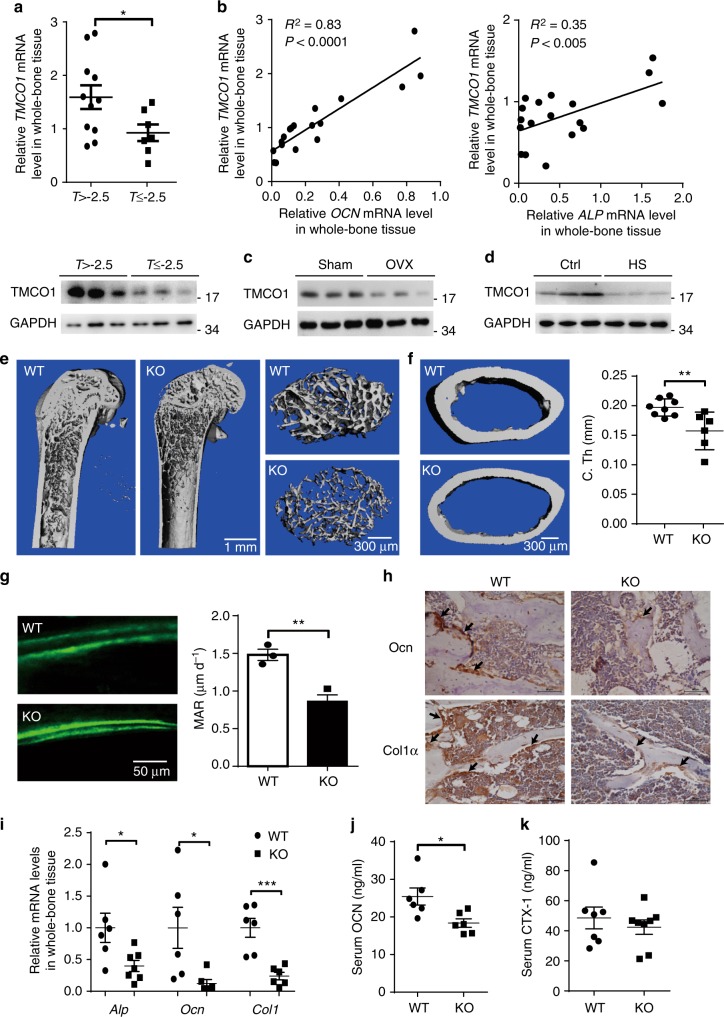
TMCO1 deficiency triggers osteoporosis. **a** T-score-associated changes in TMCO1 mRNA and protein levels in bone specimens from women with bone fracture in two T-score subgroups. **b** Correlation analysis between TMCO1 and ALP, OCN mRNA levels in bone specimens from women with bone fracture. **c** Western blot analysis of TMCO1 expression in the bone from OVX mice. **d** Western blot analysis of TMCO1 expression in the bone from mice subjected to hindlimb unloading. **e** Representative images showing the three-dimensional trabecular architecture after µCT reconstruction of the distal femurs from 2-month-old male WT (*n* = 6) and *Tmco1*^−/−^ mice (*n* = 6). Scale bars, left 1 mm; right 300 μm. **f** µCT analysis of the C.Th of distal femurs from 2-month-old male WT (*n* = 8) and *Tmco1*^−/−^ mice (*n* = 6). Scale bars, 300 μm. **g** Dual calcein-labeling and quantitative mineral apposition rate (MAR) analysis of tibia bone from WT (*n* = 3) and *Tmco1*^−/−^ mice (*n* = 3) was visualized by fluorescence micrography. Scale bars, 50 μm. Data are presented as the mean ± standard error of the mean (s.e.m.). unpaired Student’s *t* test, *P < 0.05. **h** Immunohistochemical staining of the indicated genes in tibia sections from 2-week-old *Tmco1*^−/−^ mice and WT mice. Scale bars, 50 μm. **i** Quantitative reverse transcriptase-PCR analysis of osteoblast-specific genes in bone from 2-month-old male mice. Data are presented as the mean ± s.e.m. unpaired Student’s *t* test, *P < 0.05, ***P < 0.001. **j** ELISA analysis of serum OCN (ng/mL) in 2-month-old WT (*n* = 6) and *Tmco1*^−/−^ mice (*n* = 6). Data are presented as the mean ± s.e.m. unpaired Student’s *t* test, *P < 0.05. **k** ELISA analysis of serum CTX-1 (ng/mL) in 2-month-old WT (*n* = 7) and *Tmco1*^−/−^ mice (*n* = 8). Data are presented as the mean ± s.e.m. unpaired Student’s *t* test

To explore the function of TMCO1 during bone formation, we assessed the expression of *TMCO1* in femurs from patients with fractures (Supplementary Table 1). *TMCO1* mRNA and protein levels in osteoporosis patients (T ≤ −2.5) were obviously lower than those in control patients (T > −2.5) (Fig. 1a). *TMCO1* expression was positively correlated with *ALP* (alkaline phosphatase) and *OCN* (osteocalcin) mRNA levels in these human samples (Fig. 1b). We further investigated changes in TMCO1 expression levels in bone tissues from OVX (ovariectomized) and hindlimb-unloaded (HU) mice. The results showed that decreased TMCO1 protein levels were accompanied by reduced osteoblast function in these model mice (Fig. 1c, d; Supplementary Fig. 1a, b). To identify whether the decrease in TMCO1 after OVX and unloading is caused by less expression in osteoblasts, TMCO1 expression was detected by immunofluorescent double staining. The results showed that TMCO1 expression in Col1a-positive osteoblasts after unloading and OVX was much lower than that of control, while there was no difference in OSCAR-positive osteoclasts (Supplementary Fig. 1c–f). To determine whether downregulation of TMCO1 in vivo affected bone formation, we utilized CRISPR/Cas9 technology to generate *Tmco1*-null mice that contained frameshift mutation in exon 1 of the *Tmco1* locus (Supplementary Fig. 2a). The 4 bp deletion at this location results in complete ablation of TMCO1 protein levels in mice homozygous for this *Tmco1* mutation (*Tmco1*^−/−^) (Supplementary Fig. 2b). To determine the relevance of *Tmco1* during skeletal system development, we examined the skeletons of 1-week-old *Tmco1*^−/−^ mice using Alcian blue and Alizarin red staining. Our *Tmco1*^−/−^ mice also displayed abnormal cranial facial development that was characterized by the presence of a domed skull and a shortened snout (Supplementary Fig. 2c). We observed 100% penetrance in the development of craniofacial abnormalities in *Tmco1*^−/−^ mice, the severity of the phenotype was variable in these mice during the whole growth and development stage. In our mouse model, part of neonatal pups (35.4%, 34/96) showed growth retardation and died before weaning. While those survived adult *Tmco1*^−/−^ mice had no differences in body size or weight compared with the control mice (Supplementary Fig. 2d). A micro-CT (µCT) analysis of long bones revealed that *Tmco1*^−/−^ mice exhibited dramatic losses in bone mass, thickness, and trabeculation (Fig. 1e). Trabecular bone volume (BV/TV), bone mineral density (BMD), trabecular thickness (Tb.Th), and cortical thickness (C.Th) were significantly decreased, and the trabecular spacing (Tb.Sp) was increased in *Tmco1*^−/−^ mice compared with control mice during different developmental stages (Supplementary Fig. 2e; Fig. 1f). Moreover, bone formation rates were substantially reduced in *Tmco1*^−/−^ mice compared with wild-type (WT) mice (Fig. 1g; Supplementary Fig. 2f). Meanwhile, bone histomorphometric analysis of proximal tibia from 8-week-old mice showed that the bone formation parameters, including Ob.S/BS, and N.Ob/B.Pm were decreased in *Tmco1*^−/−^ mice tibias (Supplementary Fig. 2g). Consistent with the bone deficiency phenotype, the levels of the osteoblast markers collagen type I alpha 1 (Col1a1) and osteocalcin (Ocn) were obviously reduced in 2-month-old *Tmco1*^−/−^ tibias compared with control tibias, according to immunostaining analysis (Fig. 1h). Quantitative reverse transcriptase-PCR (Q-PCR) analyses showed that the expression of osteoblast marker genes, including alkaline phosphatase (*Alp*), *Ocn*, and *Col1a1*, was significantly decreased in femurs from *Tmco1*^−/−^ mice compared with control femurs (Fig. 1i). Accordingly, the level of the bone formation marker OCN was significantly decreased in serum from *Tmco1*^−/−^ mice compared with control serum (Fig. 1j). In contrast, the levels of the bone resorption marker CTX-1, bone-related neural factors growth hormone (GH), thyroid-stimulating hormone (TSH), and neuropeptide (NPY) showed no significant changes between *Tmco1*^−/−^ mice and control mice (Fig. 1k; Supplementary Fig. 2h). Also, we observed no difference in osteoclast generation and the number of osteoclasts between WT and *Tmco1*^−/−^ mice by TRAP staining of sections from the tibias (Supplementary Fig. 3a, b). Furthermore, we detected TMCO1 protein level during the process of osteoclast differentiation and the effect of TMCO1 deficiency on osteoclast differentiation. The result showed that TMCO1 expression had no changes in the process of osteoclast differentiation (Supplementary Fig. 3c). Moreover, TRAP staining and osteoclast marker genes showed no significant changes between WT and *Tmco1*^−/−^ osteoclasts (Supplementary Fig. 3d, e). Taken together, these data suggest that TMCO1 deficiency impairs bone formation and is closely correlated with the occurrence of bone loss.

### TMCO1 regulates osteoblastogenesis through RUNX2

**Fig. 2:**
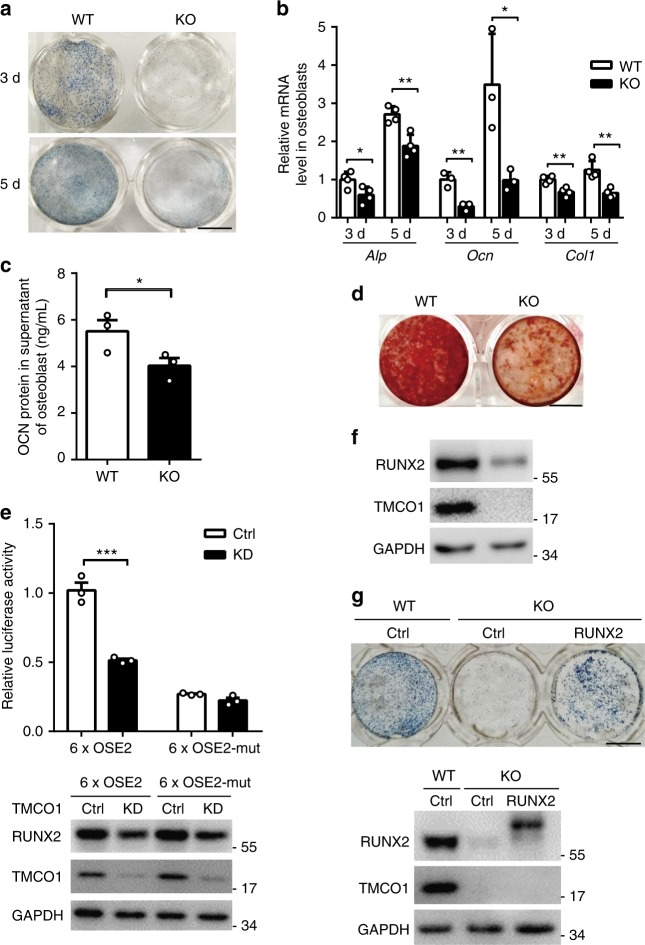
TMCO1 deficiency impairs osteoblast function. **a** Representative images of ALP staining of WT and *Tmco1*^−/−^ osteoblasts after treatment with osteoblast differentiation medium for 5 days. Scale bars, 6 mm. **b** Q-PCR analysis of osteoblast-specific genes in primary osteoblasts from newborns cultured in osteogenic medium for 3 and 5 days, *n* = 3. Representative results of three independent experiments are shown. **c** ELISA analysis of the amount of OCN protein in the supernatants of WT (*n* = 3) and *Tmco1*^−/−^ (*n* = 3) osteoblasts after treatment with the osteoblast differentiation medium for 5 days. **d** Alizarin red staining of WT and *Tmco1*^−/−^ BMSCs induced with the osteogenic medium for 21 days. Scale bars, 6 mm. **e** Measurement of RUNX2-responsive OSE2 luciferase activity in C3H10T1/2 cells transfected with control-siRNA or TMCO1-siRNA, *n* = 3. Below is the western blot analysis of RUNX2 and TMCO1 levels in the lysates. All data are presented as the mean ± s.e.m. unpaired Student’s *t* test, ***P < 0.001. Representative results of three independent experiments are shown. **f** RUNX2 protein levels in tibias from 2-month-old WT and *Tmco1*^−/−^ mice were assessed by western blotting. Representative results of three independent experiments are shown. **g** Representative images of ALP staining and western blot analysis of WT and *Tmco1*^−/−^ osteoblasts treated with an adenovirus expressing RUNX2. Scale bars, 6 mm. Representative results of three independent experiments are shown

To further explore the role of TMCO1 in osteoblastogenesis, primary osteoblast cells were isolated and cultured in the osteogenesis medium. We observed that TMCO1 levels increased during the course of osteoblast differentiation in vitro (Supplementary Fig. 4a, b). *Tmco1*^−/−^ osteoblasts displayed markedly reduced ALP activity compared with control osteoblasts (Fig. 2a). The mRNA levels of osteogenic genes, such as *Alp*, *Ocn*, and *Col1*, were significantly downregulated in *Tmco1*^−/−^ osteoblasts compared with control osteoblasts (Fig. 2b). The OCN concentration in the supernatant was also decreased in *Tmco1*^−/−^ osteoblasts compared with control osteoblasts (Fig. 2c). Furthermore, osteoblasts from BMSCs also showed a significant decrease in mineralization compared with control osteoblasts based on alizarin red staining (Fig. 2d). Similarly, impaired osteogenesis was also observed in TMCO1-knockdown MC3T3-E1 cells (Supplementary Fig. 4c–e). However, TMCO1 deficiency had no effect on osteoblast proliferation (Supplementary Fig. 4f, g). The results demonstrate that TMCO1 plays an important role in osteoblast differentiation and function.

We next sought to investigate the mechanism by which TMCO1 regulates osteogenesis in vitro. To determine the key transcription factor (TF) influenced by TMCO1, we used synthetic DNA containing a concatenated tandem array of consensus TF response elements (catTFREs) for most known TF families to enrich TFs from control and *Tmco1*-knockdown primary osteoblasts (Supplementary Fig. 5a). We succeeded in detecting 199 TFs from control osteoblasts and 93 TFs from *TMCO1*-knockdown osteoblasts. In *Tmco1*-knockdown osteoblasts, we found that 16 TFs were decreased by more than 2.5-fold compared with the corresponding control, and 6 TFs were increased by more than 1.3-fold (Supplementary Fig. 5b). Among these enriched transcription factors, RUNX2 was the most changed; the RUNX2 level in *Tmco1*-knockdown osteoblasts was only 10% that of the control. To verify the effects of TMCO1 on the RUNX2 activity in osteoblast cells, we performed a dual luciferase reporter assay using 6x OSE2-Luc specific for RUNX2 in control and *Tmco1*-siRNA knockdown C3H10T1/2 cells. TMCO1 knockdown in osteoblasts significantly reduced the RUNX2-driven activation of 6x OSE2-Luc (Fig. 2e; Supplementary Fig. 5e). Then, we verified the expression of RUNX2 in tibias from control and *Tmco1*^−/−^ mice through western blot analysis and found that RUNX2 was significantly reduced in *Tmco1*^−/−^ mice (Fig. 2f; Supplementary Fig. 5f). Accordingly, when RUNX2 was overexpressed in *Tmco1*^−/−^ primary osteoblasts, their function was obviously rescued (Fig. 2g). Although we found that RUNX2 protein levels were significantly decreased in *Tmco1*^−/−^ osteoblasts, RUNX2 mRNA levels remained unchanged (Supplementary Fig. 5c). This finding led us to explore the underlying role of TMCO1 in maintaining RUNX2 protein stability.

### Regulation of RUNX2 stability by TMCO1

**Fig. 3:**
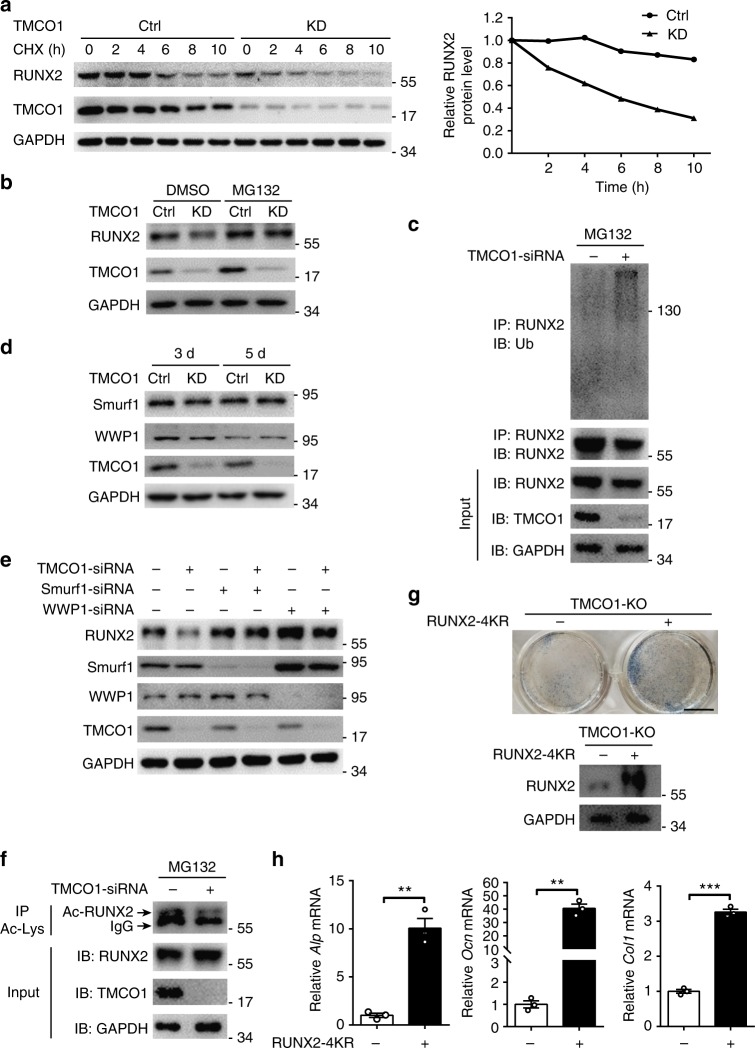
TMCO1 regualtes RUNX2 stability via promoting RUNX2 acetylation. **a** The stability of RUNX2 in TMCO1-deficient MC3T3-E1 cells was measured by treating the cells with CHX for the indicated times, followed by western blot analysis. Relative amounts of RUNX2 were calculated and shown in the graphs. Representative results of three independent experiments are shown. **b** Deficiency of TMCO1 mediates the proteasome-dependent degradation of RUNX2. Control-siRNA and TMCO1-siRNA transfected MC3T3-E1 cells were treated with 20 μM MG132 for 6 h. Protein levels of RUNX2 were measured by western blotting. Representative results of three independent experiments are shown. **c** Effect of TMCO1 on the ubiquitination of endogenous RUNX2. Control-siRNA and TMCO1-siRNA were transfected into MC3T3-E1 cells for 48 h. The cells were treated with 20 μM MG132 for 6 h, and RUNX2 ubiquitination was analyzed by western blotting using an anti-RUNX2 antibody. Experiments were successfully repeated three times. **d** Western blot analysis of the E3 ubiquitin ligase for RUNX2 in TMCO1-siRNA-transfected MC3T3-E1 cells. Representative results of three independent experiments are shown. **e** The E3 ubiquitin ligase Smurf1 is responsible for RUNX2 degradation caused by the loss of TMCO1. Control and TMCO1-deficient MC3T3-E1 cells were transfected with Wwp1 or Smurf1-siRNA for 24 h. Western blotting was used to analyze RUNX2 expression in cell lysates. Representative results of three independent experiments are shown. **f** Knockdown of TMCO1 using siRNA suppresses RUNX2 acetylation. MC3T3-E1 cells were transfected with control-siRNA and TMCO1-siRNA. The acetylation of endogenous RUNX2 was determined by IP using an anti-Ac-K antibody, followed by immunoblotting with an anti-RUNX2 antibody. The levels of endogenous RUNX2, TMCO1, and GAPDH were determined by western blotting. Experiments were successfully repeated two times. **g** Representative images of ALP staining in *Tmco1*^−/−^ osteoblasts transfected with RUNX2-4KR or not. Scale bars, 6 mm. Western blot analysis of RUNX2-4KR overexpression in *Tmco1*^−/−^ primary osteoblasts. **h** Effect of RUNX2-4KR overexpression on osteoblast-specific genes caused by TMCO1 deficiency. WT and *Tmco1*^−/−^ primary osteoblasts were transfected with RUNX2-4KR. Representative results of three independent experiments are shown. Data are presented as the mean ± s.e.m. unpaired Student’s *t* test, *n* = 3, **P < 0.01 and ***P < 0.001

RUNX2 is a critical transactivator involved in osteoblast differentiation. The stability of RUNX2 is strictly regulated during the osteogenesis process. RUNX2 has been reported to be regulated through a ubiquitin–proteasome-mediated protein degradation mechanism. To examine whether TMCO1 affects RUNX2 stability, we measured the half-life of RUNX2 by utilizing cycloheximide (CHX) treatment. The results showed that the half-life of RUNX2 dramatically decreased in the absence of TMCO1 (Fig. 3a). We next asked whether TMCO1 deficiency-mediated RUNX2 downregulation was due to ubiquitin-mediated proteasome degradation. MC3T3-E1 cells were transfected with control-siRNA or *Tmco1*-siRNA in the presence or absence of MG132, a proteasome inhibitor. Immunoblotting results demonstrated that the RUNX2 protein levels decreased in the absence of MG132 following *Tmco1*-siRNA knockdown, but remained constant in the presence of MG132 (Fig. 3b). Ubiquitination assays revealed that in *Tmco1*-siRNA knockdown MC3T3-E1 cells, polyubiquitinated RUNX2 accumulated at higher levels than in control cells (Fig. 3c). Previous studies have suggested that RUNX2 activity might be regulated through the ubiquitin–proteasome-mediated protein degradation pathway, and E3 ligases, such as Smurf1 and Wwp1, facilitate its degradation^20^. However, Smurf1 and Wwp1 protein levels showed no differences after *Tmco1* knockdown (Fig. 3d). To identify the E3 ligases that promote RUNX2 degradation in the absence of TMCO1, MC3T3-E1 cells were transfected with *Smurf1* and *Wwp1* siRNA, with or without *Tmco1*-siRNA. RUNX2 protein levels decreased in the absence of Wwp1 following *Tmco1*-siRNA knockdown, but remained constant in the absence of Smurf1 (Fig. 3e; Supplementary Fig. 5g). These results indicate that the TMCO1 deficiency-induced RUNX2 degradation was mediated by the Smurf1-dependent ubiquitin–proteasome pathway. Recent studies have indicated that acetylation can protect RUNX2 from Smurf1-mediated ubiquitination^21^; therefore, we examined whether TMCO1 regulated RUNX2 acetylation by stabilizing the RUNX2 protein. Notably, the TMCO1 deficiency resulted in the downregulation of RUNX2 acetylation levels (Fig. 3f). We transfected MC3T3-E1 cells with a RUNX2-4KR (225/230/350/351) mutant containing substitutions at all the potential acetylation sites. While WT RUNX2 was obviously downregulated after *Tmco1*-siRNA knockdown, the RUNX2-4KR mutant was extremely stable, even in the absence of TMCO1 (Supplementary Fig. 5d). Moreover, markedly reduced ALP activity in *Tmco1*^−/−^ osteoblasts could also be obviously rescued by RUNX2-4KR transfection (Fig. 3g). As expected, after overexpressed RUNX2-4KR into *Tmco1*^−/−^ primary osteoblasts, we found that the expression of *Alp, Ocn*, and *Col1a* were rescued compared with wild-type control (Fig. 3h). These data show that TMCO1 stabilizes RUNX2 through the regulation of its acetylation.

### HDAC4 is responsible for the regulation of RUNX2 by TMCO1

**Fig. 4:**
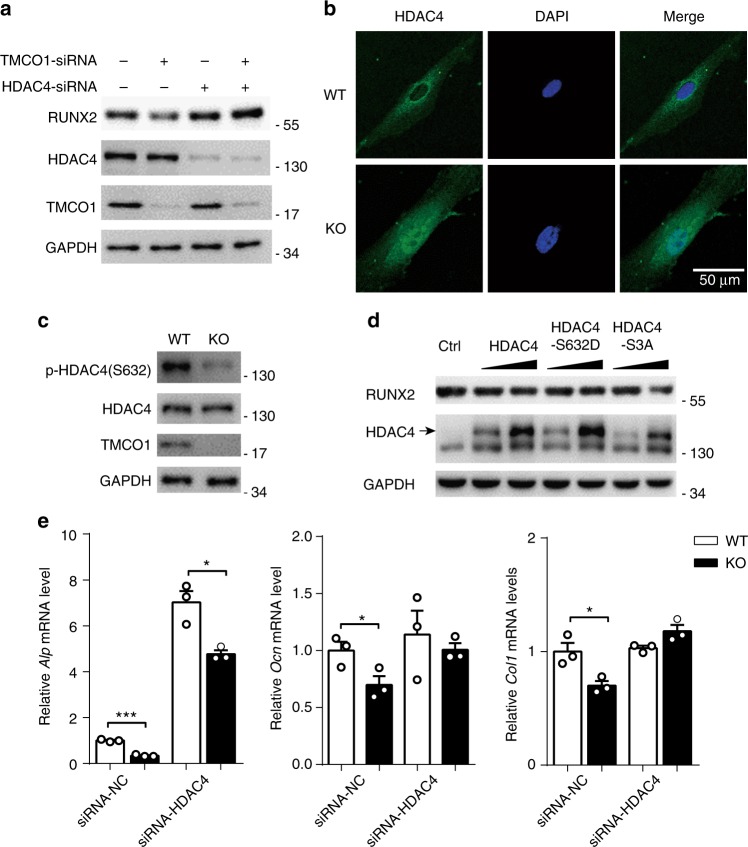
TMCO1 deficiency leads to the nuclear accumulation of HDAC4 to downregulate RUNX2. **a** HDAC4 is required for the reduction of RUNX2 in TMCO1-knockdown MC3T3-E1 cells. MC3T3-E1 cells were transfected with HDAC4-siRNA and/or TMCO1-siRNA. After 36 h, RUNX2 expression was analyzed by western blotting. Representative results of three independent experiments are shown. **b** Lack of TMCO1 promotes HDAC4 nuclear translocation in vitro. Primary osteoblasts from the calvarium in WT and *Tmco1*^−/−^ newborns were stained with an anti-HDAC4 antibody and Hoechst, followed by confocal fluorescence microscopy. Scale bars, 50 μm. Immunofluorescence assay was repeated by two independent experiments. **c** Western blot analysis of HDAC4 phosphorylation in WT and *Tmco1*^−/−^ osteoblasts. Representative results of three independent experiments are shown. **d** Effect of HDAC4 overexpression on RUNX2 protein levels in MC3T3-E1 cells. MC3T3-E1 cells were transfected with increasing amounts of HDAC4, cytoplasm-localized HDAC4-S632D, or nucleus-localized HDAC4-S3A. After 36 h, the expression of RUNX2 was analyzed by western blotting. Representative results of three independent experiments are shown. **e** Q-PCR analysis of osteoblast-specific genes in WT and *Tmco1*^−/−^ osteoblasts treated with HDAC4-siRNA. Representative results of three independent experiments are shown. Data are presented as the mean ± s.e.m. unpaired Student’s *t* test, *n* = 3, *P < 0.05, **P < 0.01, ***P < 0.001

RUNX2 acetylation is suppressed by class II HDACs, such as HDAC4^21^. HDAC^−/−^ mice exhibit premature ossification of developing bones, and overexpression of HDAC4 in proliferating chondrocytes results in phenotypes similar to those observed in *RUNX2*^−/−^ mice, such as chondrocyte hypertrophy deficiency and endochondral bone formation^22^. Based on the above-mentioned studies, we explored the role of HDAC4 in TMCO1 deficiency-induced RUNX2 degradation. We found that the inhibitory effect of TMCO1 on RUNX2 degradation was weakened when HDAC4 was knocked down in osteoblasts (Fig. 4a; Supplementary Fig. 6a). Interestingly, immunostaining results showed that TMCO1 knockout caused a significant relocation of HDAC4 from the cytoplasm to the nucleus (Fig. 4b). The same result was identified by nuclear-cytosol extraction isolation (Supplementary Fig. 6b). HDAC4 phosphorylation (S632), a key regulator of HDAC4 location, was significantly reduced in *Tmco1*^−/−^ osteoblasts (Fig. 4c). We found that HDAC4 overexpression decreased cellular RUNX2 protein levels. A phosphomimetic mutant of HDAC4, HDAC4-S632D, which localizes to the cytoplasm, failed to affect RUNX2 levels. However, the constitutively nuclear-localized HDAC4-S3A obviously decreased RUNX2 protein levels (Fig. 4d; Supplementary Fig. 6c). Accordingly, the inhibitory role of TMCO1 in osteoblast function was substantially attenuated when HDAC4 was knocked down (Fig. 4e). In summary, TMCO1 deficiency decreased HDAC4 phosphorylation and increased its accumulation in the nucleus, resulting in RUNX2 degradation.

### The essential role of CaMKII in the regulation of RUNX2

**Fig. 5:**
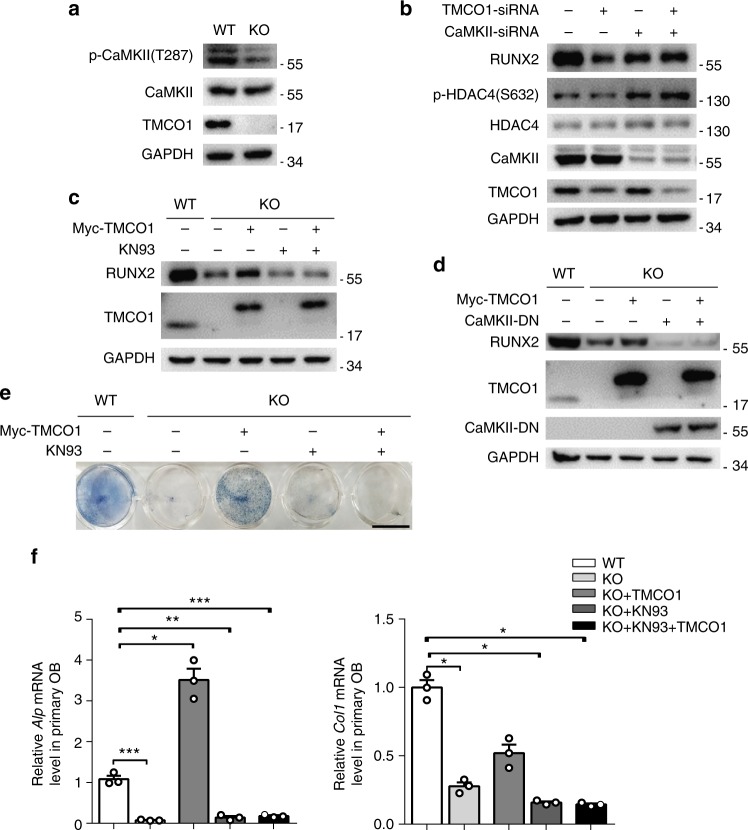
Altered CaMKII signaling in TMCO1-deficient osteoblasts. **a** Western blot analysis of CaMKII protein expression and phosphorylation in WT and *TMCO1*^−/−^ osteoblasts. Representative results of three independent experiments are shown. **b** CaMKII deficiency restricts the effect of TMCO1 knockdown on RUNX2 expression. MC3T3-E1 cells were transfected with CaMKII-siRNA and/or TMCO1-siRNA. The protein levels of RUNX2 and TMCO1 were determined by western blotting. Representative results of three independent experiments are shown. **c** Effect of KN93 on RUNX2 expression caused by TMCO1 deficiency. WT and *Tmco1*^−/−^ primary osteoblasts were infected with an adenovirus expressing TMCO1 in the presence or absence of treatment with the CaMKII inhibitor KN93. Representative results of three independent experiments are shown. **d** Effect of DN-CaMKII (dominant-negative CaMKII) on RUNX2 expression caused by TMCO1 deficiency. WT and *Tmco1*^−/−^ primary osteoblasts were infected with an adenovirus expressing TMCO1 in the presence or absence of treatment with DN-CaMKII. Representative results of three independent experiments are shown. **e** Effect of CaMKII deficiency on ALP staining caused by TMCO1 deficiency. WT and *Tmco1*^−/−^ primary osteoblasts were infected with an adenovirus expressing TMCO1 in the presence or absence of treatment with the CaMKII inhibitor KN93. Scale bars, 6 mm. **f** Effect of CaMKII deficiency on osteoblast-specific genes caused by TMCO1 deficiency. WT and *Tmco1*^−/−^ primary osteoblasts were infected with an adenovirus expressing TMCO1 in the presence or absence of treatment with the CaMKII inhibitor KN93, *n* = 3. Each group was compared with the WT. Data are presented as the mean ± s.e.m. one-way ANOVA with multiple comparison test, *P < 0.05, **P < 0.01, ***P < 0.001

HDAC4 contains a unique CaMKII docking site, which allows it to bind and respond specifically to CaMKII. Upon phosphorylation, HDAC4 is transported from the nucleus to the cytoplasm^23^. We found that CaMKII phosphorylation was significantly downregulated in *Tmco1*^−/−^ primary osteoblasts (Fig. 5a). To determine whether CaMKII is responsible for the reduced RUNX2 protein levels caused by TMCO1 deficiency, we measured changes in RUNX2 protein levels in control and TMCO1-KD MC3T3-E1 cells with or without CaMKII knockdown. In the absence of CaMKII, there were no changes in HDAC4 phosphorylation, and RUNX2 protein levels remained constant between control and *Tmco1*-knockdown cells. These results showed that CaMKII knockdown alleviated the effects of TMCO1 deficiency on RUNX2 (Fig. 5b). To determine the essential role of CaMKII in the regulation of RUNX2 stability by TMCO1, we compared the effects of TMCO1 on RUNX2 levels in the presence of the CaMKII inhibitor KN93. In *Tmco1*^−/−^ osteoblasts, the reduced RUNX2 levels were rescued by transfection with exogenous TMCO1. However, when the osteoblasts were treated with KN93, this effect was substantially suppressed (Fig. 5c). To further verify the specific effect of CaMKII on this process, osteoblasts were transfected with dominant-negative CaMKII (CaMKII-DN), a specific inhibitor of CaMKII activity. We found that the recovery effect of transfection with exogenous TMCO1 was absolutely repressed in the presence of CaMKII-DN (Fig. 5d). When CaMKII activity was inhibited, reduced *Tmco1*^−/−^ osteoblast activity could not be recovered by transfection with exogenous TMCO1, which was exhibited by the reduced ALP activity (Fig. 5e) and the decrease in *Alp* and *Col1* expression (Fig. 5f). These results demonstrate that CaMKII is required for TMCO1 to regulate RUNX2 protein stability.

### TMCO1 regulates RUNX2 dependent on local Ca^2+^ signaling

**Fig. 6:**
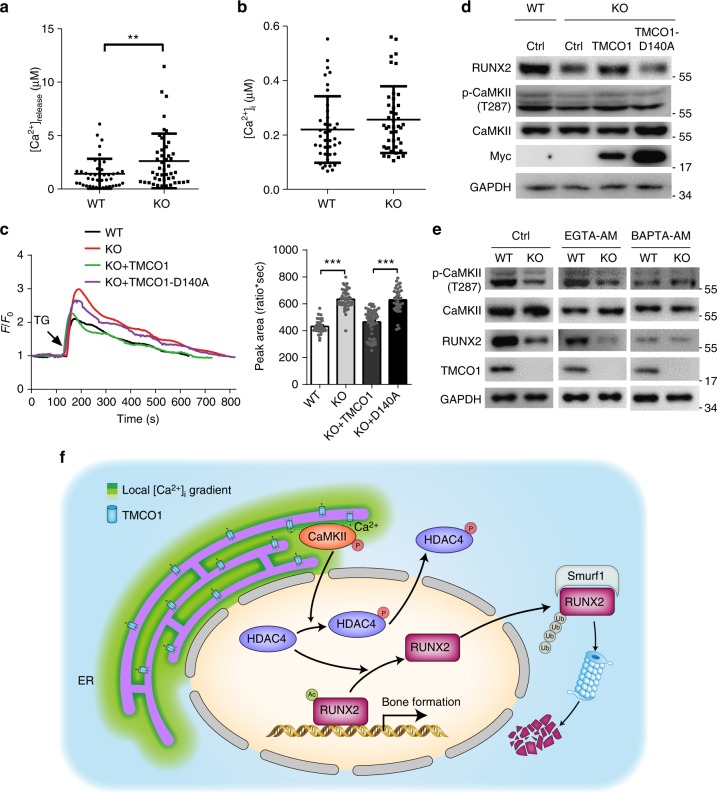
TMCO1 regulates ER Ca^2+^ load and CaMKII activation via local Ca^2+^ signaling in osteoblasts. **a** [Ca^2+^] release in primary osteoblasts from WT and *Tmco1*^−/−^ mice, *n* = 42 (WT) and *n* = 45 (KO) cells pooled from three independent experiments. **b** Resting [Ca^2+^]_i_ in primary osteoblasts from WT and *Tmco1*^−/−^ mice, *n* = 41 (WT) and *n* = 43 (KO) cells pooled from three independent experiments. Data are presented as the mean ± s.e.m. unpaired Student’s *t* test, **P < 0.01. **c** TG-induced calcium transients in WT (*n* = 29) and *Tmco1*^−/−^ (*n* = 42) osteoblasts and *Tmco1*^−/−^ osteoblasts expressing wild-type (*n* = 52) and mutated TMCO1 (*n* = 34 cells pooled across three independent experiments). Each group was compared with the WT. Data are presented as the mean ± s.e.m. one-way ANOVA with multiple comparison test, ***P < 0.001. **d** Western blot analysis of RUNX2 in WT and *Tmco1*^−/−^ osteoblasts and *Tmco1*^−/−^ osteoblasts expressing wild-type and mutated TMCO1. Representative results of three independent experiments are shown. **e** Effect of BAPTA-AM and EGTA-AM on CaMKII phosphorylation caused by TMCO1 deficiency. CaMKII protein expression and phosphorylation were analyzed by western blotting. Representative results of three independent experiments are shown. **f** Proposed functional model for the regulation of osteoblast function by TMCO1

CaMKII is well known to be activated by intracellular Ca^2+^ signaling, and TMCO1 has been identified as a conserved regulator of ER Ca^2+^ homeostasis. Therefore, we investigated TMCO1-mediated Ca^2+^ changes and their regulatory effect on osteoblasts. ER and intracellular Ca^2+^ levels were measured with the Ca^2+^-sensitive fluorescent indicator Fluo-4 AM (Supplementary Fig. 7a). The results showed that more calcium was released from the ER in *Tmco1*^−/−^ osteoblasts, suggesting that the ER Ca^2+^ store is higher in *Tmco1*^−/−^ osteoblasts than in WT cells (Fig. 6a). However, we did not observe any differences in the resting intracellular Ca^2+^ concentrations between WT and *Tmco1*^−/−^ osteoblasts (Fig. 6b). We further compared intracellular Ca^2+^ levels using Fura-2 in 1.8 mM Ca^2+^ Tyrode solution and found no differences between WT and *Tmco1*^−/−^ osteoblasts (Supplementary Fig. 7b). Next, we investigated Ca^2+^ release from the ER by inhibiting Ca^2+^ uptake with thapsigargin (TG), an inhibitor of the ER-localized Ca^2+^ ATPase (SERCA). The results showed that Ca^2+^ release from the ER was much greater and long lasting in the absence of TMCO1 than in WT cells (Supplementary Fig. 7c). We also found that Ca^2+^ transients induced by ATP, which stimulates the IP3R-mediated Ca^2+^ release, were significantly elevated in *Tmco1*^−/−^ osteoblasts compared with WT osteoblasts (Supplementary Fig. 7d). Consistently, PTH and BMP2, two important regulators of osteoblast function, also elicited enhanced Ca^2+^ release in *Tmco1*^−/−^ osteoblasts (Supplementary Fig. 7e, f). These results showed that loss of TMCO1 causes overloading of ER Ca^2+^ store and mishandling of Ca^2+^ signaling in osteoblasts, which suggested TMCO1 might work as an intercellular Ca^2+^ permeable channel. To test whether the regulation of Ca^2+^ signaling by TMCO1 is dependent on Ca^2+^ permeability, *Tmco1*^−/−^ osteoblasts were transfected with exogenous TMCO1 or a TMCO1 mutant (D140A) that lacks the Ca^2+^ permeation function. We found that the increased Ca^2+^ release after TG treatment in *Tmco1*^−/−^ osteoblasts was completely rescued by the expression of WT TMCO1, but not by the D140A mutant (Fig. 6c). Then, we analyzed the effect of TMCO1 Ca^2+^ permeation ability on osteoblast function. The results showed that WT TMCO1 rather than D140A mutant rescued RUNX2 protein levels and CaMKII phosphorylation in *Tmco1*^−/−^ osteoblasts (Fig. 6d). In addition, the mRNA levels of the osteoblast marker genes *Alp* and *Col1a* were recovered by transfection of *Tmco1*^−/−^ osteoblasts with WT TMCO1, but remained unchanged when TMCO1 D140A was exogenously expressed (Supplementary Fig. 8). The above results show that the Ca^2+^ permeability of TMCO1 is critical for the effects of TMCO1 on osteoblast function.

To further explore the underlying mechanism of decreased CaMKII activation in *Tmco1*^−/−^ osteoblasts, we treated WT and *Tmco1*^−/−^ osteoblasts with BAPTA-AM and EGTA-AM, two intracellular Ca^2+^ chelators with different kinetics, to compare the changes in CaMKII phosphorylation in these cells. Under EGTA-AM treatment, CaMKII phosphorylation and RUNX2 levels remained reduced in *Tmco1*^−/−^ osteoblasts compared with WT osteoblasts. However, BAPTA-AM abrogated the difference (Fig. 6e). BAPTA has a 100-fold faster binding rate for Ca^2+^ than EGTA and thus can reduce the local Ca^2+^ concentration more effectively^24^. This finding led us to speculate that TMCO1 may regulate CaMKII phosphorylation in a manner that is dependent on the local Ca^2+^ signal near the ER tubule surface. Here, a model was used to simulate CaMKII activation near the ER tubule surface to investigate the impact of calcium channels on the local environment. Based on previous studies, Ca^2+^ from the ER tubule can freely diffuse, bind to a buffer, and diffuse in the form of a Ca^2+^ buffer or dissociate from the buffer. The calcium channels are uniformly distributed on the tubule surface. The simulation showed that both the Ca^2+^ concentration and CaMKII activation reduced rapidly with increased distance from the ER surface. The activation of CaMKII by ER Ca^2+^ release occurred in the high [Ca^2+^] nanodomain adjacent to the ER tubule surface. Therefore, BAPTA can depress CaMKII activation by chelating nanodomain [Ca^2+^], while EGTA has no such effect (Supplementary Fig. 9). Taken together, these data strongly suggest that the nanodomain [Ca^2+^] near the ER surface is substantially decreased in *Tmco1*^−/−^ osteoblasts, resulting in reduction of CaMKII activation and its downstream signaling.

## Discussion

In this study, we found that TMCO1 is a novel regulator of bone formation and is required for osteoblast differentiation and function. *TMCO1* mRNA expression in bone tissues was obviously downregulated in osteoporosis progression, and its levels were positively correlated with the expression of osteogenic marker genes in aged postmenopausal women. These results were further verified in OVX and unloading-induced bone-loss models. TMCO1 deficiency led to reduced bone formation and osteoblast differentiation in vivo, but no significant differences were found in osteoclast function. RUNX2 was identified as the key target affected by TMCO1. RUNX2 stability was drastically reduced in *Tmco1*^−/−^ osteoblasts as a result of the upregulation of RUNX2 ubiquitination and degradation due to the downregulation of RUNX2 acetylation. The underlying mechanisms include decreased nanodomain [Ca^2+^] near the ER surface, reduced activation of CaMKII, and downregulation of HDAC4 phosphorylation. These processes result in HDAC4 enrichment in the nucleus, where it promotes RUNX2 deacetylation (Fig. 6f).

RUNX2 is the most upstream transcription factor essential for osteoblast differentiation^1,2,25^. Osteoblasts are completely absent in *Runx2*^−/−^ mice^26^. RUNX2 levels are regulated at various cellular levels, including transcriptional regulation and post-translational modifications, such as acetylation, ubiquitination, phosphorylation, and sumoylation^6,27^. Calcium is an essential signaling molecule in osteogenesis. Many hormones and cytokines, such as BMP2, vitamin D3, and PTH, have been shown to affect bone formation by modulating intracellular Ca^2+^ signaling^14,28,29^. However, little is known regarding the mechanisms by which Ca^2+^ signaling is involved in the regulation of RUNX2 activity and stability. In this study, RUNX2 was identified as the most important target of TMCO1 in bone tissues. TMCO1 deficiency promotes E3 ubiquitin ligase Smurf1-mediated RUNX2 ubiquitination and degradation. This process is mediated by reduced Ca^2+^/CaM/CaMKII signaling. In *Tmco1*^−/−^ osteoblasts, reduced CaMKII phosphorylation caused reduced HDAC4 phosphorylation, which increased HDAC4 localization in the nucleus. Therefore, the acetylation levels of RUNX2 were downregulated and ubiquitination increased. These results demonstrate the molecular link between Ca^2+^, CaMKII, HADC4, and RUNX2. The dynamic equilibrium among RUNX2 acetylation, deacetylation, and ubiquitination is maintained by TMCO1-mediated Ca^2+^ signaling.

TMCO1 is an evolutionarily conserved ER transmembrane-spanning protein that has been reported to be an ER Ca^2+^ permeable channel that responds to ER Ca^2+^ overload and regulates ER Ca^2+^ homeostasis and Ca^2+^ signaling^10^. The intracellular Ca^2+^ distribution is tightly controlled by active (ATP-dependent) calcium pumps and channels in the ER. However, little is known regarding the effect of these ER membrane proteins on osteoblast function. PANX3 is an ER Ca^2+^ channel that is required for normal progression of skeletal development and late-stage bone growth in vertebrates. PANX3 can regulate both chondrocyte and osteoblast differentiation through activation of intracellular Ca^2+^ signaling pathways^30–32^. *Panx3*-deficient osteoblasts exhibit decreased resting [Ca^2+^]_i_ levels. In *Tmco1*^−/−^ osteoblasts, we found that the ER Ca^2+^ store was significantly upregulated. However, the intracellular resting Ca^2+^ concentration remained unchanged. Surprisingly, we found that CaMKII phosphorylation was significantly reduced. BAPTA but not EGTA treatment can eliminate the differences between WT and *Tmco1*^−/−^ osteoblasts. This result indicates that the changes in the Ca^2+^ concentration and CaMKII activation occur at the nanodomain scale near the ER surface. The simulation model of CaMKII activation and the distribution of Ca^2+^ also confirmed this result. The intracellular Ca^2+^ regulates CaMKII activation, which further phosphorylates Smad and c-jun to affect osteoblast function^7,33^. Here, we determined that the TMCO1-mediated CaMKII/HDAC4 pathway can enhance RUNX2 stability and promote bone formation.

In summary, we demonstrated that TMCO1 mediates ER Ca^2+^ homeostasis in osteoblasts and plays a key role in bone formation in a manner that is dependent on the Ca^2+^ permeability of the TMCO1 channel. TMCO1 deficiency causes ER Ca^2+^ overload, and RUNX2 stability is regulated by the TMCO1–CaMKII–HDAC4 axis in a local Ca^2+^ signaling-dependent manner. Our study establishes TMCO1 as an important regulator in bone formation and provides a new therapeutic target for osteoporosis.

## Methods

### Human bone tissue preparation

Patients who had fracture caused by falling without obvious violence from the Second Affiliated Hospital of Soochow University from January 2016 to January 2017 were included in our study (inclusive criteria). Excluding criteria as below: (1) participants with fracture caused by high-energy injury and pathologic fracture due to other diseases were excluded; (2) participants with hyperparathyroidism, other congenital or acquired bone disease, history of malignancy, significant liver, or renal disease; (3) participants that had taken glucocorticoids or estrogen, or selective estrogen receptor modulators within 6 months; (4) participants that had ever taken parenteral bisphosphonates, teriparatide, calcitonin, or strontium ranelate within 12 months. Above all, we recruited 18 female patients aged 60 years or older with fracture caused by low-energy injury. The osteoporosis group was defined as a BMD T score of ≤−2.5 at the lumbar, and a T score of å −2.5 as the normal group. We obtained informed consent from the participants. All the clinical procedures were approved by the Committees of Clinical Ethics in the Second Affiliated Hospital of Soochow University (Suzhou, China).

### OVX-induced osteoporotic mouse model

All the female C57BL/6N mice used were maintained under standard animal housing conditions (12-h light, 12-h dark cycles, and free access to food and water). The mice were ovariectomized or sham-operated at 3 months of age. Twomonths after surgery (5 months of age), all the sham-operated and ovariectomized mice were euthanized for dissecting bilateral femurs. All the experimental procedures were approved by the Committees of Animal Ethics and Experimental Safety of China Astronaut Research and Training Center.

### Hindlimb-unloading mice

The hindlimb-unloading procedure was achieved by tail suspension. Briefly, the 3-month-old male C57BL/6J mice were individually caged and suspended by the tail using a strip of adhesive surgical tape attached to a chain hanging from a pulley. The mice were suspended at a 30° angle to the floor, with only the forelimbs touching the floor, this allowed the mice to move and access food and water freely. The mice were subjected to hindlimb unloading through tail suspension for 28 days. After killing, bilateral femurs and tibiae were dissected and processed for microCT examination, bone histomorphometry analysis, and real-time PCR analysis. All the experimental procedures were approved by the Committees of Animal Ethics and Experimental Safety of China Astronaut Research and Training Center.

### Mice

*Tmco1*^−/−^ mice were generated by CRISPR/Cas9. SgRNA targeting the sequence GTGCACCGCGCTGCTCGCCGAGG (underscored, PAM) on exon 1 of mouse *Tmco1* was designed with the online tool (http://crispr.mit.edu/)^34^ and inserted into the BbsI site of the pX330 vector (Addgene catalog no. 42230) to generate px330-sg*TMCO1*. T7 promoter was added to Cas9 and sgRNA coding region by PCR amplification. T7-Cas9 and T7-sgRNA PCR product were gel purified and used as the template for in vitro transcription (IVT) using RiboMAX Large Scale RNA Production Systems (Promega). Both the Cas9 mRNA and the sgRNAs were purified using MEGAclear kit (Life Technologies) and eluted in RNase-free water. B6D2F1 (C57BL/6 X DBA2) female mice strains were used as embryo donors, and RNA samples were used for one-cell embryo injection to produce the offspring at Tsinghua University Laboratory Animal Research Center. The primers employed for TMCO1 genotyping primers were 5′-GTCCCGCCACGTCTCT-3′ and 5′- AGTGAGGCTCCCGATC-3′. The gene disruption was ultimately validated by Sanger’s sequencing. All mice were bred and maintained under specific pathogen-free conditions. All the experimental procedures were approved by the Committees of Animal Ethics and Experimental Safety of China Astronaut Research and Training Center.

### Measurement of serum OCN and CTX-1 concentrations

We determined serum concentrations of OCN using the OCN ELISA kit (R&D) according to the instructions provided. The levels of serum CTX-1 were measured using the mouse CTX-1 ELISA kit (Sangon Biotech).

### Assessment of new bone formation

For analysis of bone formation in vivo, 2-month-old mice were injected intraperitoneally with calcein (20 mg/kg body weight) in a time sequence of 10 and 3 days before being killed. Tibias were harvested, cleared of soft tissue, fixed in 70% ethanol. Undecalcified sections were cut at a thickness of 5 µm and examined using fluorescence microscopy.

### RNA extraction and real-time PCR

The total RNA from bone tissues or cells was extracted with TRIzol reagent (Invitrogen) as per the manufacturer’s instructions. The cell samples were washed with PBS before being treated with TRIzol. RNA (0.5 μg) was reverse transcribed with PrimeScript RT reagent Kit (Takara, China) according to the manufacturer’s instructions. Real-time PCR was performed using SYBR Premix Ex Taq II Kit (Takara, China) to detect expression of osteogenic genes. Gapdh was used as a normalization control for mRNA using the 2-△△CT method.

Mouse primers used in this study: *Gapdh*, forward: 5′-A A C A T C A A A T G G G G T G A G G C C-3′ and reverse: 5′-G T T G T C A T G G A T G A C C T T G G C-3′; *Alp*, forward: 5′-A T C T T T G G T C T G G C T C C C A T G-3′ and reverse: 5′-T T T C C C G T T C A C C G T C C A C-3′; *Bglap*, forward: 5′-C C A A G C A G G A G G G C A A T A-3′ and reverse: 5′-T C G T C A C A A G C A G G G T C A-3′; *Col1α1*, forward: 5′-G G G A C C A G G A G G A C C A G G A A G T-3′ and reverse: 5′-G G A G G G C G A G T G C T G T G C T T T-3′; *Runx2*, forward: 5′-C A A G A A G G C T C T G G C G T T T A-3′ and reverse: 5′-T G C A G C C T T A A A T G A C T C G G-3′; *Tmco1*, forward: 5′-T G A T G G T A G A G T G G T G G C A A A G-3′ and reverse: 5′-G G T G G T G G G C C A A G A A A T C C-3′.

Human primers: *GAPDH*, forward: 5′-A C A A C T T T G G T A T C G T G G A A G G-3′ and reverse: 5′-G C C A T C A C G C C A C A G T T T C-3′; *TMCO1*, forward: 5′-T G T T T G C T A T T G G C T T T T G T-3′ and reverse: 5′-A G T C T G T G G T G T C A T C T C C C-3′.

### Micro-computed tomography (Micro-CT) analysis

High-resolution micro-CT analyses were performed on the distal femurs from each mouse using a model of μ40 Scanco (SCANCO Medical, Bruttisellen, Switzerland). In the femurs, the trabecular bone proximal to the distal growth plate was selected for analyses within a conforming volume of interest (cortical bone excluded) commencing at a distance of 840 μm from the growth plate and extending a further longitudinal distance of 1680 μm in the proximal direction. Cortical measurements were performed in the diaphyseal region of the femur starting at a distance of 3.57 mm from the growth plate and extending a further longitudinal distance of 210 μm in the proximal direction. All trabecular bone from each selected slice was segmented for three-dimensional reconstruction to calculate the following parameters: BMD, BV/TV, Tb.Th, and Tb.Sp.

### Skeletal whole-mount staining

Skeletal preparations were double stained with alcian blue and alizarin red S (ARS)^35^. After mice were skinned, eviscerated and fixation with 95% ethanol for 3 days, the samples were transferred into acetone for an additional 48-h incubation. Skeletal preparations were stained for 3 days in Alcian blue solution. After staining, the samples were washed three times for 30 min in 95% EtOH followed by treatment of 2% KOH for 3–4 h. After stained with ARS solution for 3–4 h, skeletons were cleared in 1% KOH/20% glycerol and dehydrated in 95% EtOH overnight.

### Histological analysis

The tibia of mice were fixed with 4% paraformaldehyde for 48 h followed by decalcification in 10% EDTA for 3–4 weeks, and 6 -μm sections were prepared on a rotation microtome. Paraffin-embedded sections were deparaffinized in xylene and rehydrated. The samples were stained with hematoxylin–eosin and TRAP (Sigma) according to the standard protocol.

For immunohistochemical staining, sections were deparaffinized in xylene and rehydrated. Antigen retrieval was performed with protease K at 37 °C for 15 min. A solution of 3% H_2_O_2_ was used to block the activity of endogenous peroxidase. The sections were then incubated overnight at 4 °C with rabbit polyclonal antibody to osteocalcin (1:100, 23418-1-AP, Proteintech) and Col1a (1:400, ab96723, Abcam). After three times washes in PBS, biotinylated secondary antibodies were then added and incubated for 1 h at room temperature, followed by color development with DAB kit (ZSGB-bio). Negative control experiments were done by omitting the primary antibodies. The sections were examined using a microscope (ECLIPSE Ci-S, Nikon).

### Cell culture

Human embryonic kidney cell line (HEK293A) were purchased from NICLR (National Infrastructure of Cell Line Resource, Beijing, China) and cultured with the high-glucose minimum essential medium (Dulbecco’s modified Eagle’s medium) containing 10% fetal bovine serum (FBS). Mouse MSC cell line C3H10T1/2 were purchased from NICLR (Beijing, China) and cultured with the high-glucose minimum essential medium (Dulbecco’s modified Eagle’s medium) containing 10% fetal bovine serum (FBS). MC3T3-E1 cells were purchased from NICLR (Beijing, China) and maintained in the minimum essential medium (MEM) alpha containing 10% FBS. For the culture of osteoblast lineage cells, calvariae from newborn mice were dissected aseptically and treated with 0.1% collagenase and 0.2% dispase. The isolated cells were maintained in the minimum essential medium (MEM) alpha containing 10% FBS^36^. For osteoblast differentiation, primary osteoblasts were cultured in α-MEM containing 10% FBS and 10 nM dexamethasone (Sigma), 50 µg/ml of ascorbic acid, and 5 mM β-glycerophosphate.

### Plasmids, siRNAs, and adenovirus

To generate the Myc-Runx2 construct, the full-length Runx2 open-reading frame was amplified from the total cDNA of mice and cloned into the pCMV-Myc vector containing Myc-tag. The point mutant construct (K225R, K230R, K350R, and K351R) was generated by site-directed mutagenesis. Full-length and truncated of HDACs plasmids were provided by Dr. Eric Olson (UT Southwestern, USA).

The control, mouse-specific *Tmco1, Wwp1, Smurf1, Hdac4*, and *Camk2d* siRNAs were purchased from GenePharma.

mouse *Tmco1* siRNA sequences: 5′-UUAAACAUUCCCAUUAAGGCA-3′

mouse *Wwp1* siRNA sequences: 5′-GCAGAGAAAUACUGUUUAU-3′

mouse *Smurf1* siRNA sequences: 5′-UUAAACAUUCCCAUUAAGGCA-3′

mouse *Hdac4* siRNA sequences: 5′-UUAAACAUUCCCAUUAAGGCA-3′

mouse *Camk2d* siRNA sequences: 5′-UCUAGAAUCUGUUGUAUACAA-3′.

For adenoviral expression, the human TMCO1 and mouse RUNX2 CDS were cloned into pAd/CMV/V5-DEST vector (Life Technologies) following the manufacturer’s instructions. The TMCO1-D140A mutant was generated from the wild-type TMCO1 construct (Myc-tagged) by point mutation using site-directed mutagenesis kit (Takara). Ad-CaMKII-DN (in which Lys43 of CaMKII-δC was replaced with alanine (K43A)) was a gift from Dr. Yan Zhang (Peking University, China). The adenovirus were produced by transfecting adenoviral vector into HEK293A cells and purified using Vivapure AdenoPACK™ 20 RT kit (Sartorius).

### Alkaline phosphatase and Alizarin red staining

Alkaline phosphatase staining was monitored using a Vector Blue Substrate Kit (SK-5300; Vector Laboratories). According to the protocol, MC3T3-E1 or primary osteoblast cells were incubated with the substrate working solution for 20–30 min. The whole procedure was protected from light. After 2 min of rinsing in deionized water, slides were treated with Mayer’s hematoxylin solution for 10 min.

Cells were fixed in 70% ice-cold ethanol for 15 min and rinsed with double-distilled H_2_O. Cells were stained with 40 mM Alizarin red S (Sigma), pH 4.0, for 15 min with gentle agitation. Cells were rinsed five times with double-distilled H_2_O and then rinsed for 15 min using 1 × PBS with gently agitating.

### catTFRE pull-down

Transfected cells were washed thrice with phosphate-buffered saline, then suspended in 600 μl of cytoplasmic extraction reagent I (CER I) buffer (NE-PER kit, Thermo Scientific). Nuclear proteins were extracted in accordance with the manufacturer’s instructions. Protein concentrations were determined using the Bradford method (Bio-Rad SmartSpec Plus, Bio-Rad Laboratories, Inc., USA). Two milligrams of control or KD NEs were used for catTFRE pull-down and trypsin digestion^37^. Then the samples from catTFRE in-gel digestion were analyzed by LC-MS/MS.

### IP and immunoblotting

Cells were lysed in lysis buffer (50 mM Tris, pH 7.5, 250 mM NaCl, 0.1% sodium dodecyl sulfate, 2 mM dithiothreitol, 0.5% NP-40, 1 mM PMSF and protease inhibitor cocktail) on ice for 30 min. Protein fractions were collected by centrifugation at 15,000 *g* at 4 °C for 30 min and then separated by 10% SDS–PAGE followed by western blotting according to a standard protocol. The membranes were blocked with 5% bovine serum albumin and incubated with specific antibodies overnight. A horseradish peroxidase-labeled secondary antibody was added and visualized using an Enhanced Chemiluminescence Kit (Millipore). Antibodies used were: TMCO1 (1:500, Sigma, AV49429), Wwp1 (1:500, Sigma, SAB2102717), RUNX2 (1:1000, Cell Signaling Technology, #8486), HDAC4 (1:1000, Cell Signaling Technology, #5392), p-HDAC4 (1:1000, S632, Cell Signaling Technology, #3424), Myc-Tag (1:1000, Cell Signaling Technology, #2276), CaMKII (1:1000, GeneTex, GTX111401), p-CaMKII (1:1000, T287, GeneTex, GTX52342), Smurf1 (1:1000, Abcam, ab38866), GAPDH (1:5000, Abclonal Technology, AC033). Western blots were reproduced by three independent experiments.

For immunoprecipitations, cells were lysed in lysis buffer (0.5% NP-40, 10% glycerol, 1 mM EGTA, 1 mM EDTA, 150 mM NaCl, 50 mM Tris, pH 7.8) with protease inhibitor cocktail (Roche Applied Science) at 4°C for 30 min and lysates were collected by centrifugation (14,000 × *g*, 15 min). Lysates were subjected to IP with Anti-Ac-K-100 (1:100, Cell Signaling Technology, #9814) at 4 °C overnight, then protein A/G (Santa Cruz) was added into cell lysates for 2 h. Followed by washing with lysis buffer, cell lysates were subjected to SDS–PAGE electrophoresis and immunoblotting with the indicated antibody.

All uncropped images of blots are shown in Supplementary Fig. 10.

### Ubiquitylation assay

For detection of endogenous ubiquitylation of RUNX2, siRNA-transfected MC3T3-E1 cells were treated with the proteasome inhibitor MG132 (20 µM; Sigma) for 6 h, and proteins were immunoprecipitated with the RUNX2 antibody (2 µg, #8486S, Cell Signaling Technology), followed by immunoblotting with anti-ubiquitin antibody (1:1000, Cell Signaling Technology, #3936).

### Transient transfection and luciferase reporter assay

C3H10T1/2 cells were seeded at 2 × 10^4^ cells/well into 24-well plate, allowed to settle overnight, then transfected using lipofectamine 3000 with a luciferase reporter plasmid (6X OSE2) and pRL-TK (Promega) along with various combinations of expression plasmids as indicated. Reporter plasmid with binding-region mutations was used as a control. After 48 h, the cells were lysed with passive lysis buffer (Promega), and lysates were used for the dual luciferase reporter assay (Promega) according to the manufacturer’s instructions. Luminescent signals were quantified by luminometer (Glomax, Promega), and each value from the firefly luciferase construct was normalized by *Renilla* luciferase assay. All luciferase experiments were repeated at least three times.

### Immunofluorescence staining and colocalization analysis

After transfected with plasmid and/or adenovirus, cells grown on confocal dish were fixed with 4% paraformaldehyde for 15 min, washed twice with PBST (PBS + 0.5% Tween 20), and permeated with 0.5% Triton X-100 for 25 min. Cells were blocked in 1% BSA, 0.1% Triton X-100 in PBS for 1 h at 37 °C. Cell samples were incubated with relevant primary antibodies against HDAC4 (1:100, 17449-1-AP, Proteintech) and Myc-Tag (1:200, # 2272 S, Cell Signaling Technology) overnight. After extensive washes with PBST, cells were incubated with TRITC or FITC-conjugated anti-mouse IgG or anti-rabbit IgG (ZSGB-Bio) antibodies. Cells were counterstained with 5 μg/ml 4, 6-diamidino-2-phenylindole (DAPI) in PBS and then mounted onto the slides in antifading solution containing 0.25% DABCO. Images were acquired using Nikon ECLIPSE Ci-S epifluorescence microscope.

### Intracellular Ca^2+^ measurement

Cells were seeded on 25-mm glass cover slips and cultured with the α-MEM medium. For Ca^2+^ measurement, the coverslip was transferred to chamber and cells were loaded with 5 μM fluo-4, AM (Molecular Probe) for 20 min at 37 °C in Tyrode solution: 137 mM NaCl, 20 mM HEPES (Sigma), 10 mM glucose (Sigma), 1.2 mM MgCl_2_•6H_2_O (Sigma), 1.2 mM NaH_2_PO_4_•2H_2_O (Sigma), and 5.4 mM KCl (Sigma), pH 7.35. Cells were then rinsed twice with Tyrode solution and mounted on the inverted stage of a confocal scope (Zeiss LSM 710). Fluorescence excitation was performed using 488 -nm laser, and detection filters were set at 530 nm. Images were acquired every 3 s and analyzed using Interactive Data Language (IDL, Research Systems) software. For testing of resting [Ca^2+^]_i_ and [Ca^2+^]_i_ released from the ER, cells were scanned for 20–30 s to obtain F_resting_, then replaced the solution with 0 Ca^2+^ Tyrode solution including 4 mM EGTA (Invitrogen), 5 μM thapsigargin (Molecular probes), and 10 μM A23187 (Sigma). Stored calcium was released to the cytoplasm immediately. We defined the peak value as F_ER release_. Added 100 μM BAPTA, AM (Molecular probes) into solution to obtain F_min_. Then replaced the solution with 10 mM Ca^2+^, 5 μM thapsigargin, 12 μM A23187 (Sigma), 3 μM FCCP (Sigma), and 20 mM 2-DG (Sigma) in Tyrode solution. The stable value was F_max_. Finally, [Ca^2+^]_i_ was calibrated using the equation [Ca^2+^] = Kd × (F–F_min_)/(F_max_–F). The method is modified from Grzegorz Grynkiewicz^38^.

### [Ca^2+^] and CaMKII activity profile

Here, we bring up a simple simulation of CaMKII activation near the ER tubule surface to investigate the impact of calcium leak on local environment. Calcium from the ER tubule can freely diffuse, bind on the buffer and diffuse as the form of calcium buffer, or dissociate from buffer. The calcium leaks are uniformly distributed on the tubule surface. [Ca^2+^] and buffers near the tubule are depicted by the following ODES:

1 $$\left\{ {\begin{array}{*{20}{c}} {\frac{{\partial \left[ {{\mathrm{Ca}}^{2 + }} \right]}}{{\partial {\mathrm{t}}}} = D_{{\mathrm{Ca}}}\nabla ^2\left[ {{\mathrm{Ca}}^{2 + }} \right] + {J}_{{\mathrm{BA}}} + {J}_{{\mathrm{EG}}} + {J}_{{\mathrm{EN}}}} \\ {\frac{{\partial \left[ {{\mathrm{CaEN}}} \right]}}{{\partial {t}}} = {D}_{{\mathrm{CaEN}}}\nabla ^2\left[ {{\mathrm{CaEN}}} \right] - {J}_{{\mathrm{EN}}}} \\ {\frac{{\partial \left[ {{\mathrm{CaEG}}} \right]}}{{\partial t}} = {D}_{{\mathrm{CaEG}}}\nabla ^2\left[ {{\mathrm{CaEG}}} \right] - {J}_{{\mathrm{EG}}}} \\ {\frac{{\partial \left[ {{\mathrm{CaBA}}} \right]}}{{\partial {t}}} = D_{{\mathrm{CaBA}}}\nabla ^2\left[ {{\mathrm{CaBA}}} \right] - {J}_{{\mathrm{BA}}}} \end{array}} \right.$$

where [Ca^2+^] refers to the free calcium concentration, [CaEN], [CaEG], and [CaBA] refer to binding endogenous buffer, EGTA, and BAPTA concentration. *J*_EN_, *J*_EG_, and *J*_BA_ refer to the reaction flux between buffers and calcium.

$$\begin{array}{l}J_{{\mathrm{EN}}} = - {{{\rm{kEN}}_{{\rm{on}}}}} \times \left( {{\mathrm{EN}}_{{\mathrm{tot}}} - \left[ {{\mathrm{CaEN}}} \right]} \right) \times \left[ {{\mathrm{Ca}}^{2 + }} \right] + {{{\rm{kEN}}_{{\rm{off}}}}} \times \left[ {{\mathrm{CaEN}}} \right]\\ J_{{\mathrm{BA}}} = - {{{\rm{kBA}}_{{\rm{on}}}}} \times \left( {{\mathrm{BA}}_{{\mathrm{tot}}} - \left[ {{\mathrm{CaBA}}} \right]} \right) \times \left[ {{\mathrm{Ca}}^{2 + }} \right] + {{{\rm{kBA}}_{{\rm{off}}}}} \times \left[ {{\mathrm{CaBA}}} \right]\\ J_{{\mathrm{EG}}} = - {{{\rm{kEG}}_{{\rm{on}}}}} \times \left( {{\mathrm{EG}}_{{\mathrm{tot}}} - \left[ {{\mathrm{CaEG}}} \right]} \right) \times \left[ {{\mathrm{Ca}}^{2 + }} \right] + {{{\rm{kEG}}_{{\rm{off}}}}} \times \left[ {{\mathrm{CaEG}}} \right]\end{array}$$

This is an axisymmetric problem, $\nabla ^2 = \frac{{\partial ^2}}{{\partial r^2}} + \frac{1}{r}\frac{\partial }{{\partial r}}$.

The initial condition is as follows:

$$\begin{array}{l}r = r_{{\mathrm{tube}}},J_{{\mathrm{Ca}}} = - D_{{\mathrm{Ca}}}\frac{{d\left[ {{\mathrm{Ca}}^{2 + }} \right]}}{{{\mathrm{dr}}}},\\ \quad J_{{\mathrm{CaEN}}} = \frac{{d\left[ {{\mathrm{CaBA}}} \right]}}{{{\mathrm{dr}}}} = 0,J_{{\mathrm{CaEG}}} = \frac{{d\left[ {{\mathrm{CaBA}}} \right]}}{{{\mathrm{dr}}}} = 0,J_{{\mathrm{CaBA}}} = \frac{{d\left[ {{\mathrm{CaBA}}} \right]}}{{{\mathrm{dr}}}} = 0;\\ r \to \infty ,\left[ {{\mathrm{Ca}}^{2 + }} \right]_\infty = 0.1{\mathrm{\mu}} {\mathrm{M}},\\ \quad \left[ {{\mathrm{CaEN}}} \right]_\infty = \frac{{{\mathrm{EN}}_{{\mathrm{tot}}}\left[ {{\mathrm{Ca}}^{2 + }} \right]_\infty }}{{\left[ {{\mathrm{Ca}}^{2 + }} \right]_\infty + K_{{\mathrm{dEN}}}}},\left[ {{\mathrm{CaEG}}} \right]_\infty = \frac{{{\mathrm{EG}}_{{\mathrm{tot}}}\left[ {{\mathrm{Ca}}^{2 + }} \right]_\infty }}{{\left[ {{\mathrm{Ca}}^{2 + }} \right]_\infty + K_{{\mathrm{dEG}}}}},\left[ {{\mathrm{CaBA}}} \right]_\infty = \frac{{{\mathrm{BA}}_{{\mathrm{tot}}}\left[ {{\mathrm{Ca}}^{2 + }} \right]_\infty }}{{\left[ {{\mathrm{Ca}}^{2 + }} \right]_\infty + K_{{\mathrm{dBA}}}}};\end{array}$$

CaMKII activity can be calculated by the Hill equation^39^:

2 $${\mathrm{CaMKII}}_{\mathrm{a}} = \frac{{\frac{{\left[ {{\mathrm{Ca}}^{2 + }} \right]^{4.4}}}{{Kd\_{\mathrm{Ca}}}}}}{{1 + \frac{{\left[ {{\mathrm{Ca}}^{2 + }} \right]^{4.4}}}{{Kd\_{\mathrm{Ca}}}}}}$$

Parameters used in the simulation are listed in Table 1.

**Table 1:** Model parameters

Parameters	Value	Units	Description	References
*D*_Ca_	300	μm^2^/*s*	Diffusion coefficient of free calcium	–
*D*_CaEN_	15	μm^2^/s	Diffusion coefficient of CaEN	^40^
*D*_CaEG_	220	μm^2^/s	Diffusion coefficient of CaEG	^40^
*D*_CaBA_	220	μm^2^/s	Diffusion coefficient of CaBA	^40^
*EN*_tot_	50	μM	endogenous buffer concentration	–
*kEN*_on_	100	μm^−1^/s	Rate constant for binding	^40^
*kEN*_off_	5000	/s	Rate constant for dissociation	^40^
*K*_dEN_	50	μM	Dissociation constant of buffer	^40^
*BA*_tot_	100	μM	BAPTA concentration	–
*kBA*_on_	400	μm^−1^/s	Rate constant for binding	^40^
*kBA*_off_	88	/s	Rate constant for dissociation	^40^
*K*_dBA_	0.22	μM	Dissociation constant of buffer	^40^
*EG*_tot_	100	μM	EGTA concentration	–
*kEG*_on_	2.5	μm^−1^/s	Rate constant for binding	^40^
*kEG*_off_	0.45	/s	Rate constant for dissociation	^40^
*K*_dEG_	0.18	μM	Dissociation constant of buffer	^40^
*I*	0.1	ρA/μM^2^	Calcium current	–
*r*_tube_	30	nm	Radius of ER tubule	^40^
*t*_limit_	330	nm	Upper limit of integration	–
*n*_H_	4.4	Dimensionless	Hill coefficient	^39^
*Kd_*Ca	1.6^4.4^	μM^4.4^	Apparent dissociation constant	^39^
[Ca^2+^]_∞_	0.1	μM	Calcium concentration in cytosol	–

The model was simulated on an DELL desktop PC with an Intel i7-4790 CPU at 3.60 GHz and 8.0 GB ofca (The MathWorks).

The average [Ca^2+^] and CaMKII activity along [*r*_tube_, *r*_limit_] can be gotten from two integrations (Table 2):

3 $$\left[ {{\mathrm{Ca}}^{2 + }} \right]_{{\mathrm{average}}} = \frac{{\mathop {\int}\limits_{{\boldsymbol{r}}_{{{{\mathrm{tube}}}}}}^{{\boldsymbol{r}}_{{\mathrm{{{limit}}}}}} {2{\boldsymbol{\pi r}}\left[ {{\mathrm{{{Ca}}}}^{2 + }} \right]{\mathrm{{{dr}}}}} }}{{{\boldsymbol{\pi }}\left( {{\boldsymbol{r}}_{{\mathrm{{{infinity}}}}}^2 - {\boldsymbol{r}}_{{\mathrm{{{limit}}}}}^2} \right)}}$$

4 $${\mathrm{CaMKII}}_{{\mathrm{a}}\_{\mathrm{average}}} = \frac{{\mathop {\int}\limits_{{\boldsymbol{r}}_{{{{\mathrm{tube}}}}}}^{{\boldsymbol{r}}_{{{{\mathrm{limit}}}}}} {2{\boldsymbol{\pi r}}{\mathrm{CaMKII}}_{\mathrm{a}}\left( {\left[ {{{{\mathrm{Ca}}}}^{2 + }} \right]} \right){{{\mathrm{dr}}}}} }}{{{\boldsymbol{\pi }}\left( {{\boldsymbol{r}}_{{{{\mathrm{infinity}}}}}^2 - {\boldsymbol{r}}_{{{{\mathrm{limit}}}}}^2} \right)}}$$

**Table 2:** Integration results of endogenous buffer, EGTA addition, and BAPTA addition

Parameter	$[ {{\mathrm{Ca}}^{2 + }} ]_{{\mathrm{average}}}\,( {{\mathrm{\mu}} {\mathrm{M}}} )$	CaMKII_a*_*average_	$\frac{{{\mathrm{CaMKII}}_{{\mathrm{a}}\_{\mathrm{average}}}}}{{[ {{\mathrm{CaMKII}}_{\mathrm{a}}} ]_{{\mathrm{cyto}}}}}{\mathrm{as}}$	$\frac{{{\mathrm{CaMKII}}_{{\mathrm{a}}\_{\mathrm{average}}}}}{{{\mathrm{CaMKII}}\_{\mathrm{EN}}_{{\mathrm{a}}\_{\mathrm{average}}}}}$
Endogenous buffer	0.1802	7.6230 × 10^−5^	15.14	1
EGTA addition	0.1748	6.7082 × 10^−5^	13.33	0.88
BAPTA addition	0.1096	8.3150 × 10^−6^	1.652	0.11

Endogenous buffer: EN_tot_ = 50 μM

TA addition: EN_*tot*_ = 50 μM, EG_tot_ = 100 μM

BAPTA addition: EN_tot_ = 50 μM, BA_tot_ = 100 μM

[Ca^2+^]_∞_ = 0.1 μM, [CaMKII_a_]_∞_ = 5.0335 × 10^−6^

### Statistical analysis

For statistical analysis, all quantitative data are presented as the mean ± s.e.m. Data were generated from several independently replicates. Statistical differences among groups were analyzed by one-way analysis of variance (ANOVA) with a post hoc test to determine group differences in the study parameters. All statistical analyses were performed with Prism software (GraphPad prism for windows, version 6.0, Nashville, TN, USA). Statistical significance was evaluated using either unpaired Student’s *t* test or one-way ANOVA for analyzing multiple samples. Differences were considered significant at *P < 0.05, **P < 0.01, ***P < 0.001.

### Reporting summary

Further information on experimental design is available in the Nature Research Reporting Summary linked to this article.

## Supplementary information

Supplementary Information

Reporting Summary

## Data Availability

All relevant data are available from the corresponding author upon reasonable request.
