# Supplementary material for: TMCO1-mediated Ca2+ leak underlies osteoblast functions via CaMKII signaling
Source: Nat Commun. 2019 Apr 8;10:1589. doi: 10.1038/s41467-019-09653-5 (PMC6453895; doi:10.1038/s41467-019-09653-5)
Supplement: Supplementary file 1 — Supplementary Information [file 41467_2019_9653_MOESM1_ESM.pdf]

# TMCO1-mediated $Ca^{2+}$ leak underlies osteoblast functions via CaMKII signaling

Li et al.

## Supplementary Figure 1

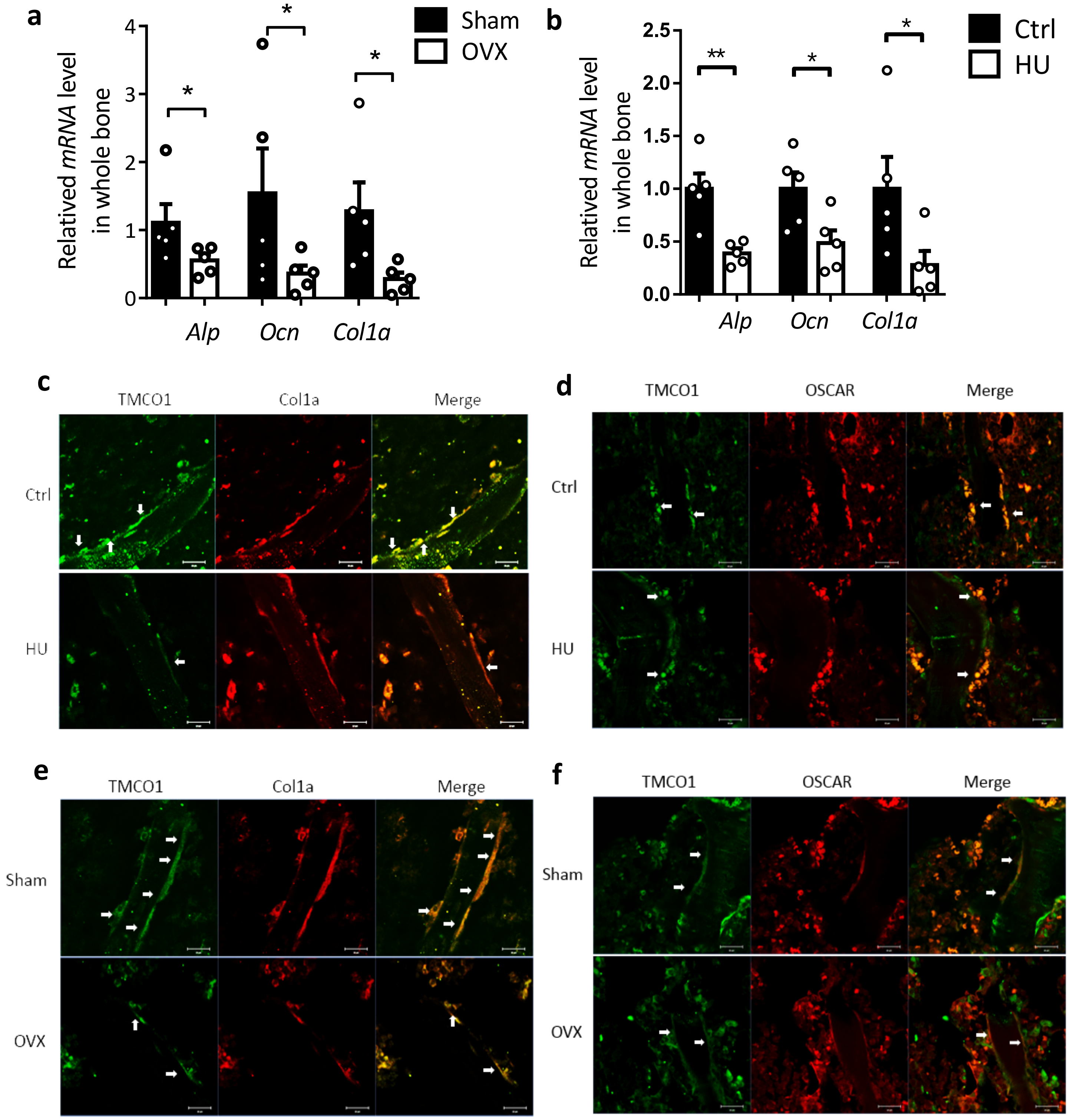

**Supplementary Figure 1. Osteogenic genes expression in osteoporosis mouse models.** (a) Real-time PCR analysis of *Alp, Ocn* and *Clo1a* mRNA levels in whole bone after surgery (sham, n = 5; OVX, n = 5). (b) Real-time PCR analysis of *Alp, Ocn* and *Clo1a* mRNA levels in whole bone collected from HU28 mice (n = 5) and age-matched control mice (n = 5). Data are presented as the mean ± s.e.m. unpaired Student's t-test, *P < 0.05, **P < 0.01 and ***P < 0.001. (c) Tibias from Hindlimb unloading (HU) and control mice were stained with an anti-TMCO1 antibody, and anti-Col1a antibody, followed by confocal fluorescence microscopy. (d) Tibias from Hindlimb unloading (HU) and control mice were stained with an anti-TMCO1 antibody, and anti-OSCAR antibody, followed by confocal fluorescence microscopy. Scale bars, 20 μm. (e) Tibias from OVX and control mice were stained with an anti-TMCO1 antibody, and anti-Col1a antibody, followed by confocal fluorescence microscopy. Scale bars, 20 μm. (f) Tibias from OVX and control mice were stained with an anti-TMCO1 antibody, and anti-OSCAR antibody, followed by confocal fluorescence microscopy. Scale bars, 20 μm.

# Supplementary Figure 2

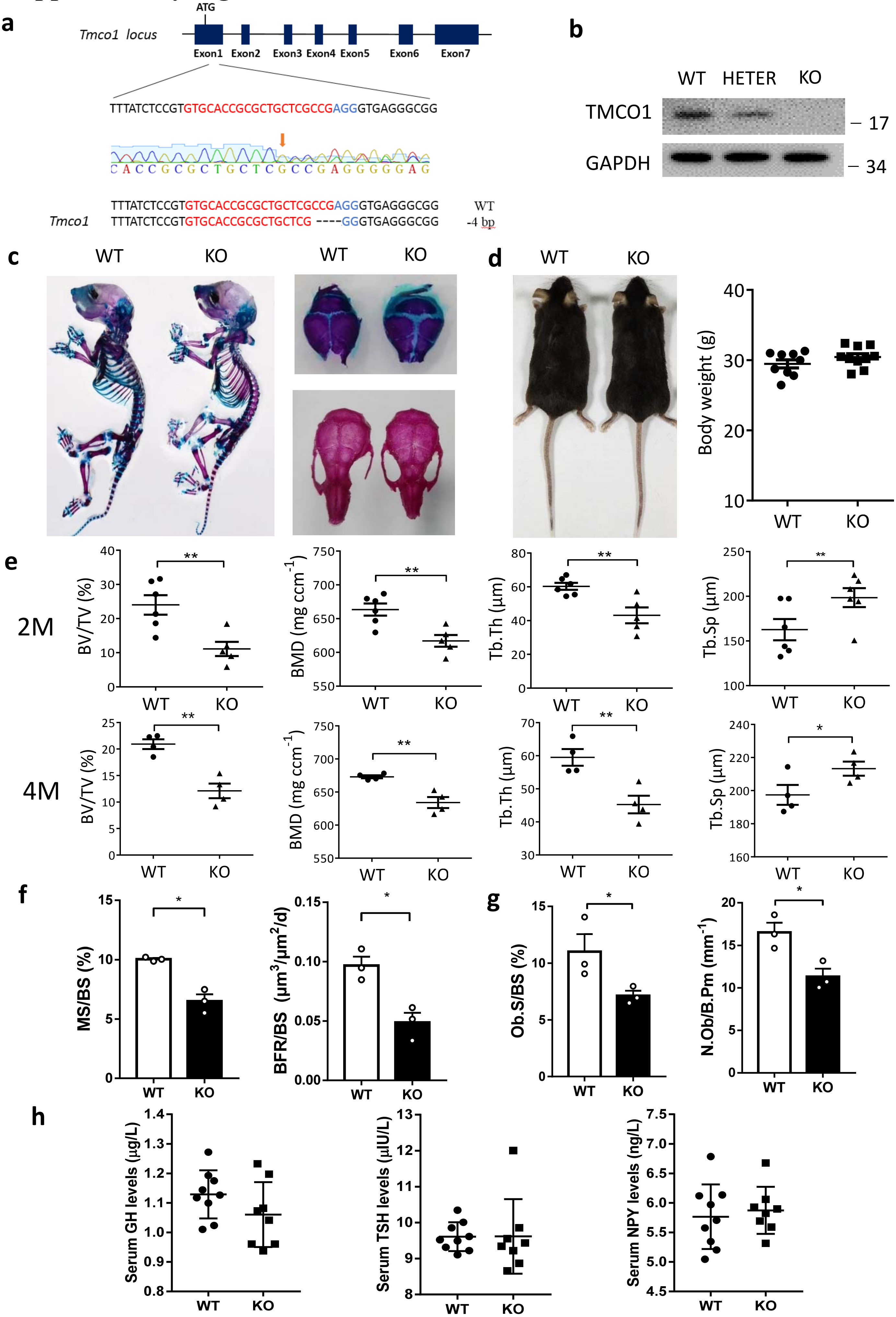

**Supplementary Figure 2. Reduced bone formation with normal body weight in *Tmco1*$^{-/-}$ mice.** (a) Illustration of the TMCO1 frameshift mutation generated in mice with CRISPR/Cas9. (b) Western blot analysis showing that the TMCO1 protein is absent in total protein from *Tmco1*$^{-/-}$ tibia. (c) Left, Alizarin red and alcian blue staining of 1-week-old and WT and *Tmco1*$^{-/-}$ mice; upper right, Alizarin red and alcian blue staining of skulls from 1-week-old and WT and *Tmco1*$^{-/-}$ mice; bottom right, Alizarin red staining of skulls from 3-month-old and WT and *Tmco1*$^{-/-}$ mice. (d) Representative images and weights of male WT and *Tmco1*$^{-/-}$ mice at 4 months of age. (e) Upper, quantitative μCT analysis of distal femurs from 2-month-old WT ($n$ = 6) and KO mice ($n$ = 5), including BV/TV, BMD, Tb.Th and Tb.Sp. Data are presented as the mean ± s.e.m. unpaired Student's t-test, *$P$ < 0.05 and **$P$ < 0.01. Bottom, quantitative μCT analysis of distal femurs from 4-month-old WT ($n$ = 4) and KO mice ($n$ = 4), including BV/TV, BMD, Tb.Th and Tb.Sp. (f) Bone formation rate per bone surface (BFR/BS) and mineralizing surface/bone surface (Ob.S/BS) of the proximal tibia from the groups of mice indicated. n = 3 in each group. (g) Osteoblast number/bone perimeter (N.Ob/B.Pm) and osteoblast surface/bone surface (Ob.S/BS) of the proximal tibia from the groups of mice indicated. n = 3 in each group. (h) Related neural factors detection in serum of WT mice (n = 9) and Tmco1$^{-/-}$ mice (n = 8). GH, Growth Hormone. TSH, Thyroid Stimulating Hormone. NPY, neuropeptide. Data are presented as the mean ± s.e.m. unpaired Student's t-test, *$P$ < 0.05, **$P$ < 0.01.

## Supplementary Figure 3

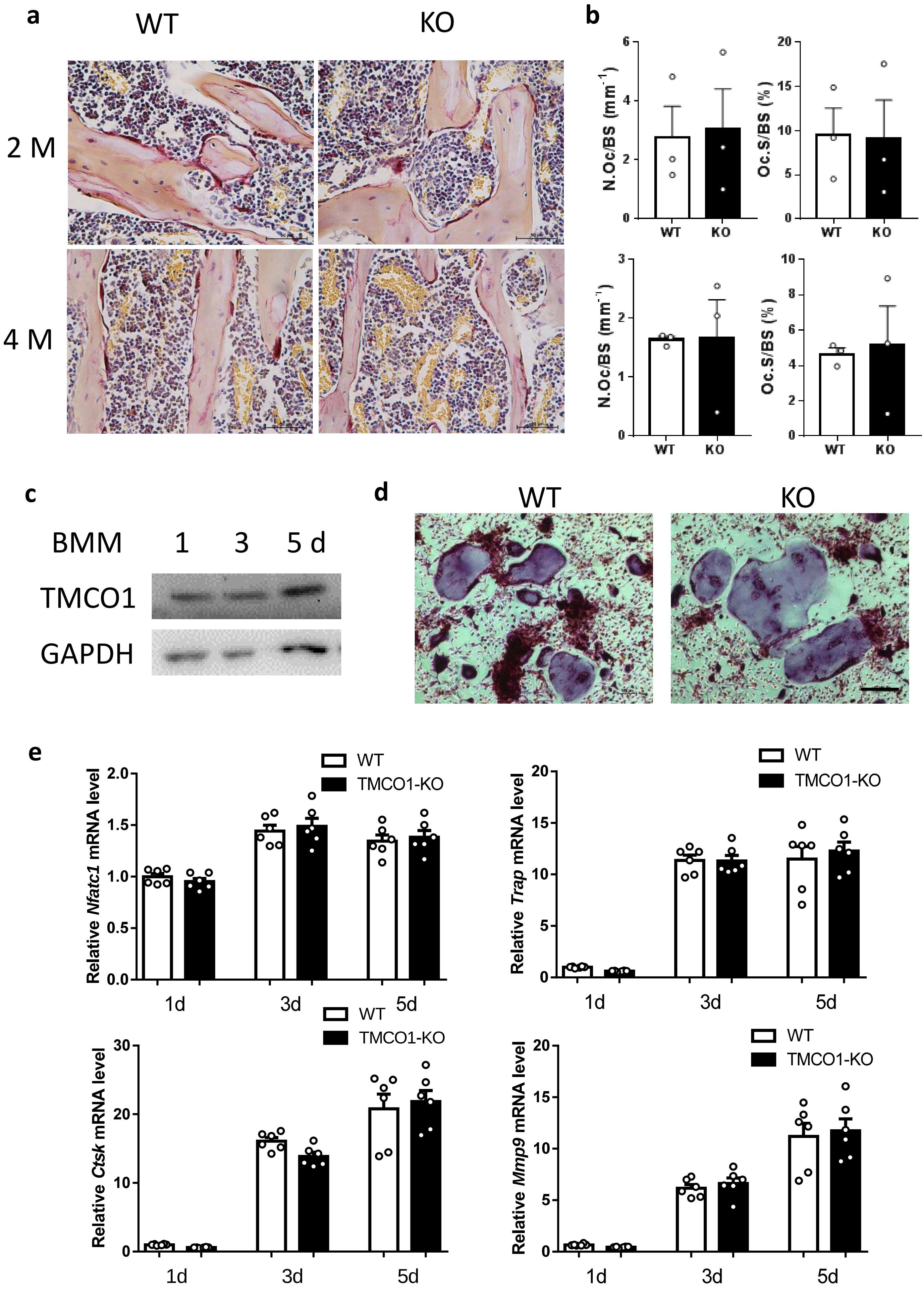

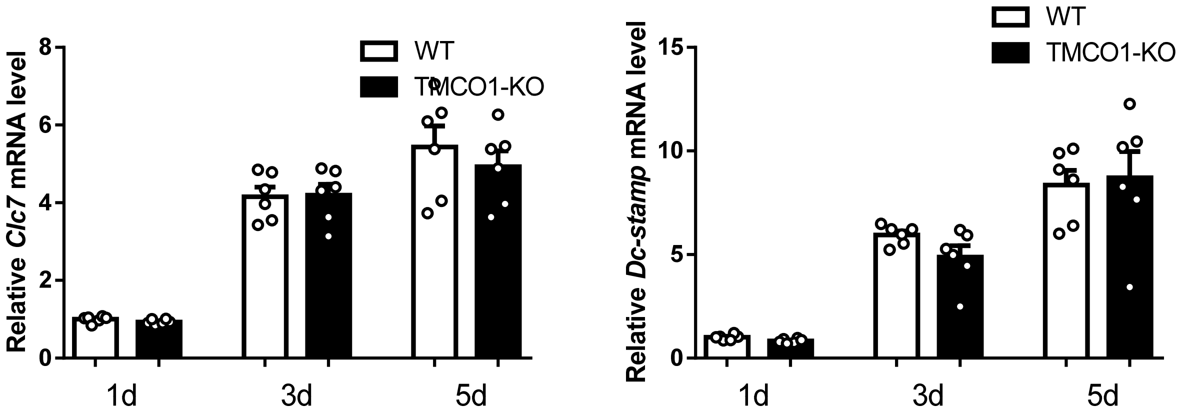


**Supplementary Figure 3. Effect of TMCO1 deficiency on osteoclast function.** (a) Histological sections of tibias from 2-month-old and 4-month-old male WT and *Tmco1*$^{-/-}$ mice stained for tartrate-resistant acid phosphatase (TRAP). Scale bars, 50 µm. (b) Osteoclast number/bone surface (N.Oc/BS) and osteoclast surface/bone surface (Oc.S/BS) of the proximal tibia from the groups of mice indicated. n = 3 in each group. (c) Analysis of TMCO1 protein levels during osteoclast differentiation. Representative results of three independent experiments are shown. (d) Representative images of TRAP staining of WT and *Tmco1*$^{-/-}$ osteoclast induced with osteoclastogenesis medium for 5 days. Representative results of three independent experiments are shown. Scale bars, 200 µm. (e) Q-PCR analysis of osteoclast-specific genes in WT and *Tmco1*$^{-/-}$ osteoclast treated with osteoclastogenesis medium for 1, 3 or 5 days. Representative results of three independent experiments are shown. Data are presented as the mean ± s.e.m. unpaired Student's t-test, *P < 0.05, **P < 0.01 and ***P < 0.001.

# Supplementary Figure 4

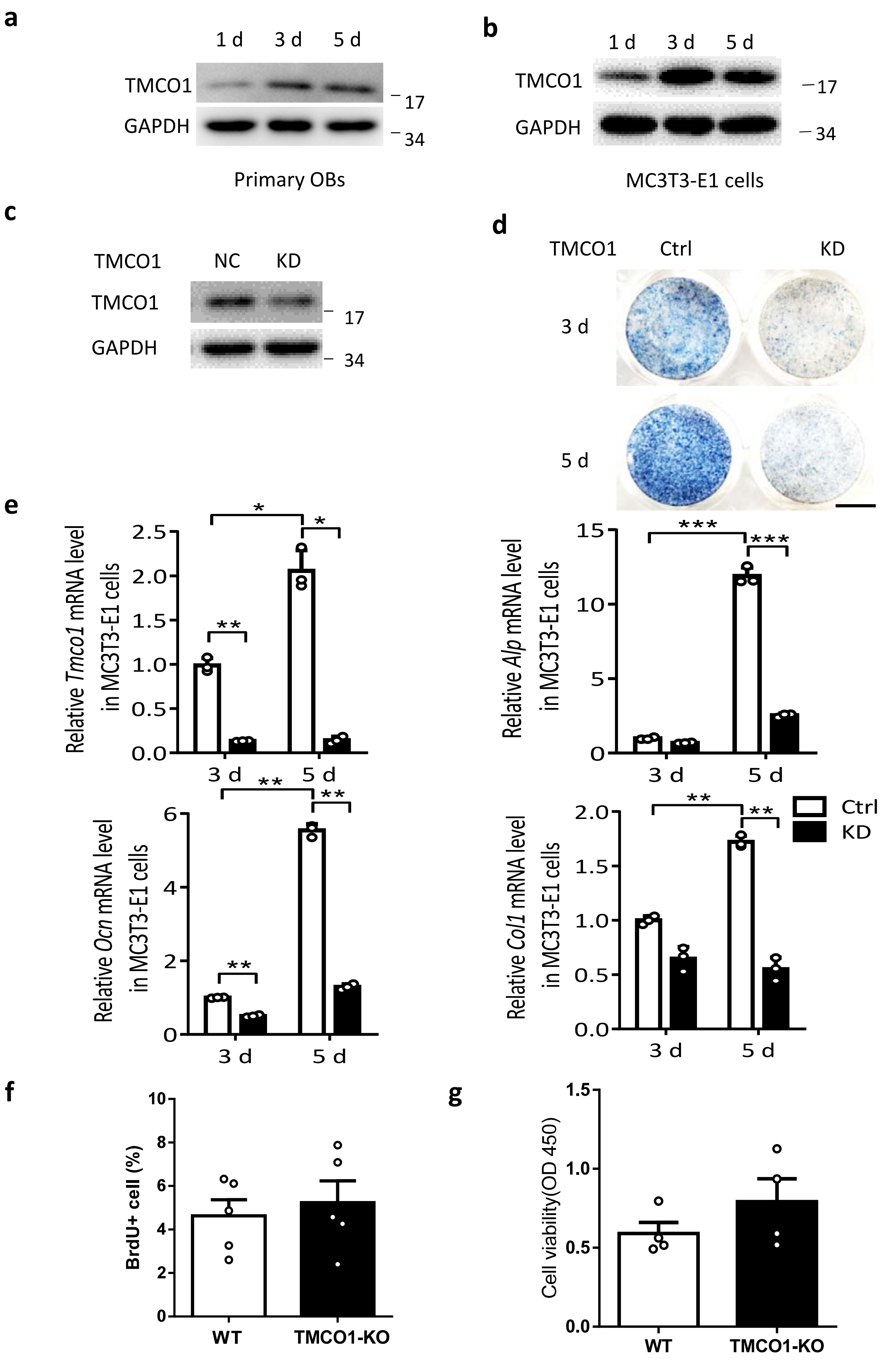

**Supplementary Figure 4. TMCO1 knockdown inhibits osteoblast differentiation.** (a) Analysis of TMCO1 protein levels during osteoblast differentiation in primary osteoblasts. Representative results of three independent experiments are shown. (b) Analysis of TMCO1 protein levels during the process of osteogenic differentiation in MC3T3-E1 cells. Representative results of three independent experiments are shown. (c) The protein levels of TMCO1 in primary osteoblasts were assessed by western blotting after the cells were transfected with control-siRNA or TMCO1-siRNA. Representative results of three independent experiments are shown. (d) Representative images of ALP staining of control and TMCO1-knockdown MC3T3-E1 cells after treatment with osteoblast differentiation medium for 3 or 5 days. Scale bars, 6 mm. (e) Q-PCR analysis of osteoblast-specific genes in control and TMCO1-knockdown MC3T3-E1 cells treated with osteogenic medium for 3 or 5 days. Representative results of three independent experiments are shown. (f) $Tmco1^{-/-}$ and WT primary osteoblasts were incubated with BrdU for 2 h and then subjected to FACS analysis. (g) Osteoblast cell viability was detected by CCK-8 assays in $Tmco1^{-/-}$ and WT primary osteoblasts. Representative results of three independent experiments are shown. Data are presented as the mean ± s.e.m. one-way ANOVA with multiple comparison test, $^{*}P < 0.05$, $^{**}P < 0.01$ and $^{***}P < 0.001$.

## Supplementary Figure 5

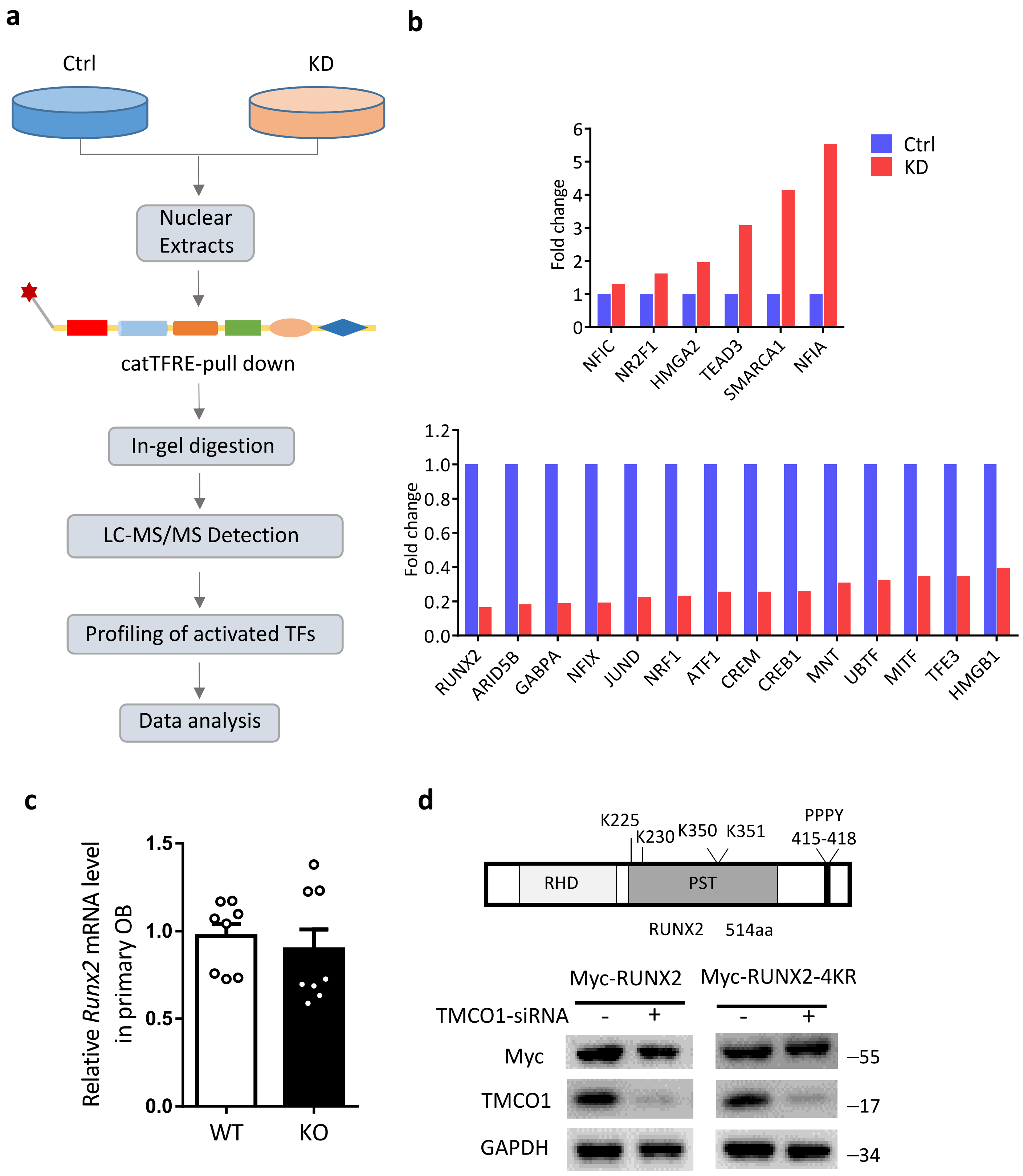

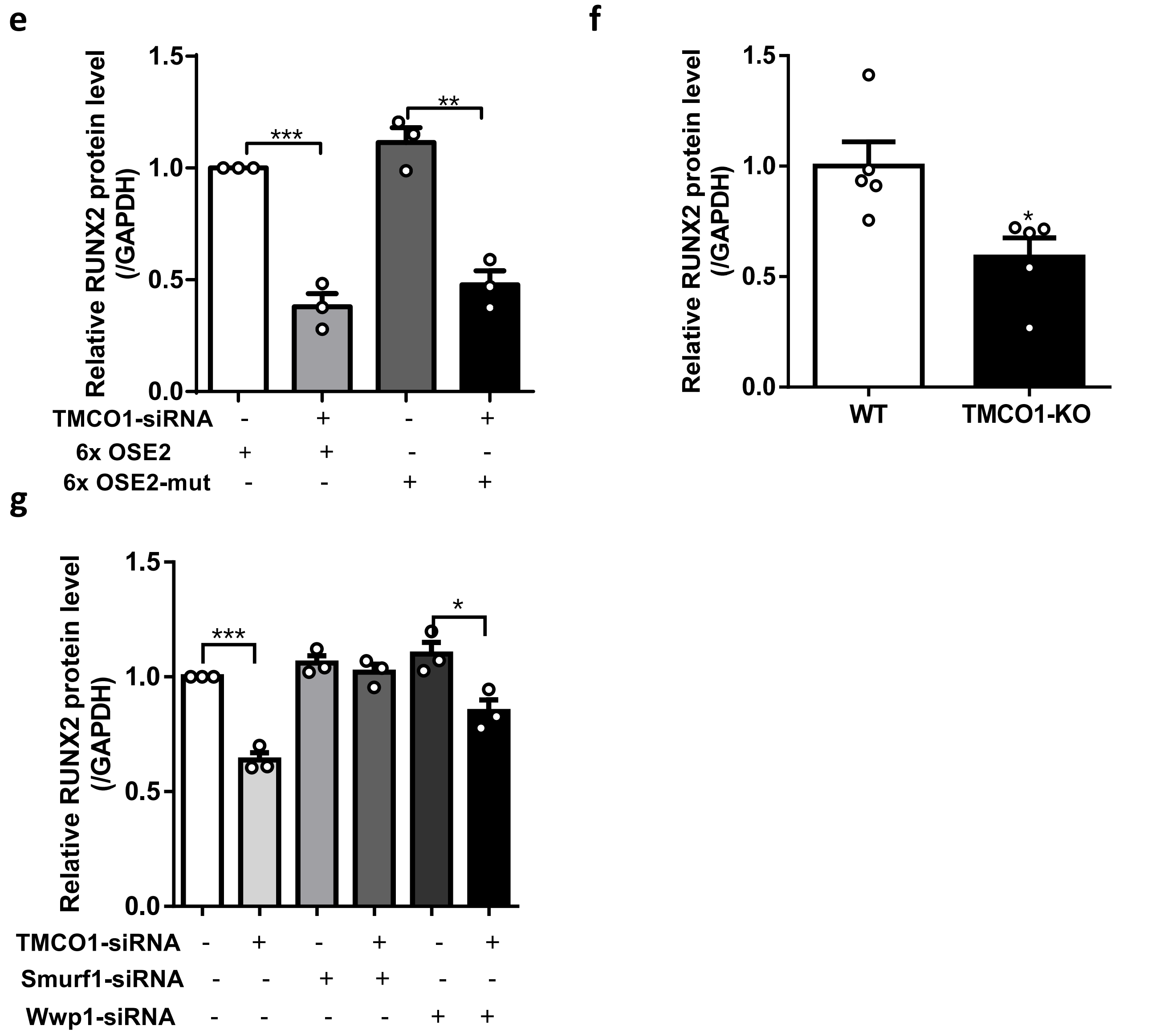


**Supplementary Figure 5. Profiling of key transcription factor regulated by TMCO1.** (a) Experimental strategy for catTFRE analysis. Control and TMCO1-siRNA knockdown primary osteoblasts were collected to extract nuclear extracts for catTFRE enrichment, and MS data were subjected to further bioinformatics analysis. (b) Identification of TF components regulated by TMCO1 inactivation. The upper panel shows the upregulated TFs in TMCO1-knockdown cells, and the lower panel shows the downregulated TFs. (c) Q-PCR analysis of RUNX2 expression in WT and *Tmco1*$^{-/-}$ osteoblasts treated with osteogenic medium for 5 days. Representative results of three independent experiments are shown. (d) A RUNX2 mutant containing lysine to arginine (KR) substitutions is stable and resistant to degradation caused by the knockdown of TMCO1. Representative results of three independent experiments are shown. (e) Quantitative analysis of RUNX2 levels for Figure 2e. (f) Quantitative analysis of RUNX2 levels for Figure 2f. (g) Quantitative analysis of RUNX2 levels for Figure 3e. Data are presented as the mean ± s.e.m. unpaired Student's t-test, *P < 0.05, **P < 0.01 and ***P < 0.001.

# Supplementary Figure 6

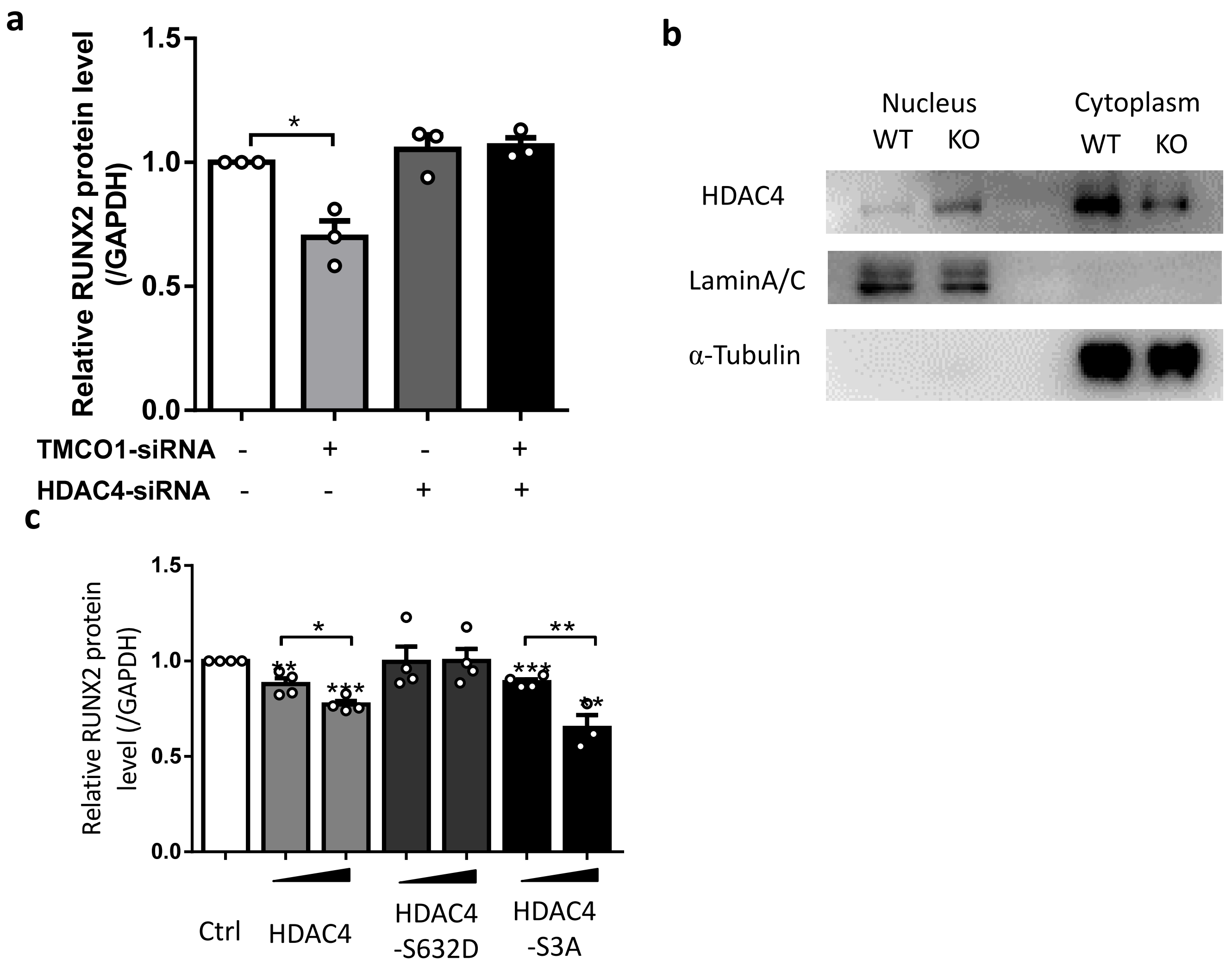


**Supplementary Figure 6. TMCO1 deficiency leads to the nuclear accumulation of HDAC4 to downregulate RUNX2 level.** (a) Quantitative analysis of RUNX2 levels for Figure 4a. (b) Analysis of nuclear and cytoplasm HDAC4 expression in WT and *Tmco1*$^{-/-}$ osteoblast. Representative results of three independent experiments are shown. (c) Quantification of RUNX2 protein level in MC3T3-E1. Data are presented as the mean ± s.e.m. one-way ANOVA with multiple comparison test,, **P* < 0.05, **P < 0.01 and ***P < 0.001.

# Supplementary Figure 7

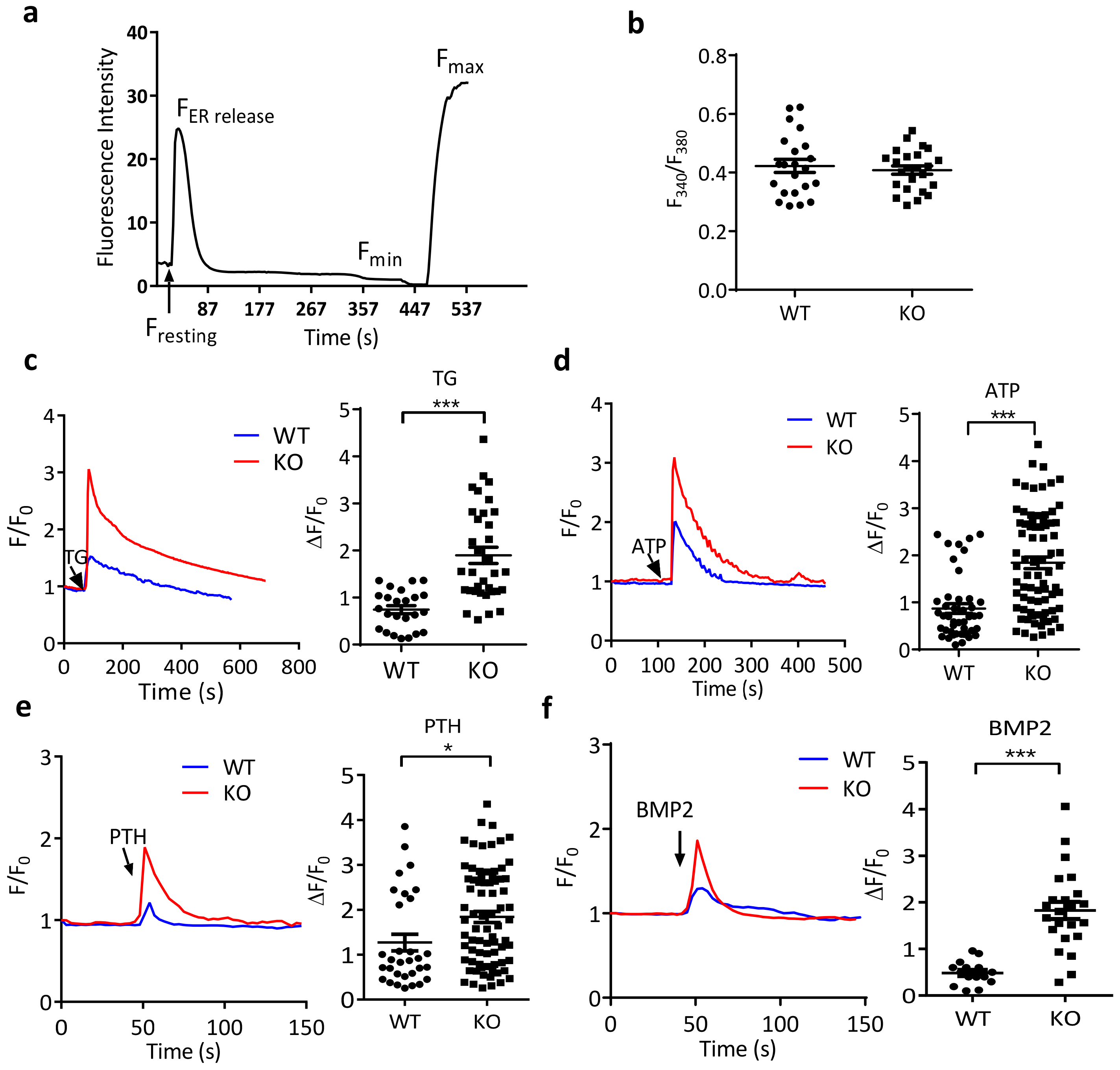

**Supplementary Figure 7. Effects of TMCO1 deficiency on calcium signaling in osteoblasts.** (a) $Ca^{2+}$ measurement of resting $[Ca^{2+}]_i$ and $[Ca^{2+}]$ released from the ER in *Tmco1*$^{-/-}$ osteoblasts by imaging cellular $Ca^{2+}$ signals with Fluo-4, AM. (b) Cytosolic $Ca^{2+}$ levels in WT and *Tmco1*$^{-/-}$ primary osteoblasts. The cytosolic $Ca^{2+}$ concentration was represented by the 340/380 ratio of Fura-2. (c) TG-evoked calcium transients in tWT and *Tmco1*$^{-/-}$ osteoblasts in 1.8 mM $Ca^{2+}$ Tyrode's solution. The amplitude of calcium transients was represented by △F/F0. (d) ATP-evoked calcium transients in WT and *Tmco1*$^{-/-}$ osteoblasts in 1.8 mM $Ca^{2+}$ Tyrode's solution. (e) PTH-triggered calcium transients in WT and *Tmco1*$^{-/-}$ primary osteoblasts in 1.8 mM $Ca^{2+}$ Tyrode's solution. (f) BMP2-triggered calcium transients in WT and *Tmco1*$^{-/-}$ primary osteoblasts in 1.8 mM $Ca^{2+}$ Tyrode's solution. Data are presented as the mean ± s.e.m. unpaired Student's t-test, *P < 0.05 and ***P < 0.001.

## Supplementary Figure 8

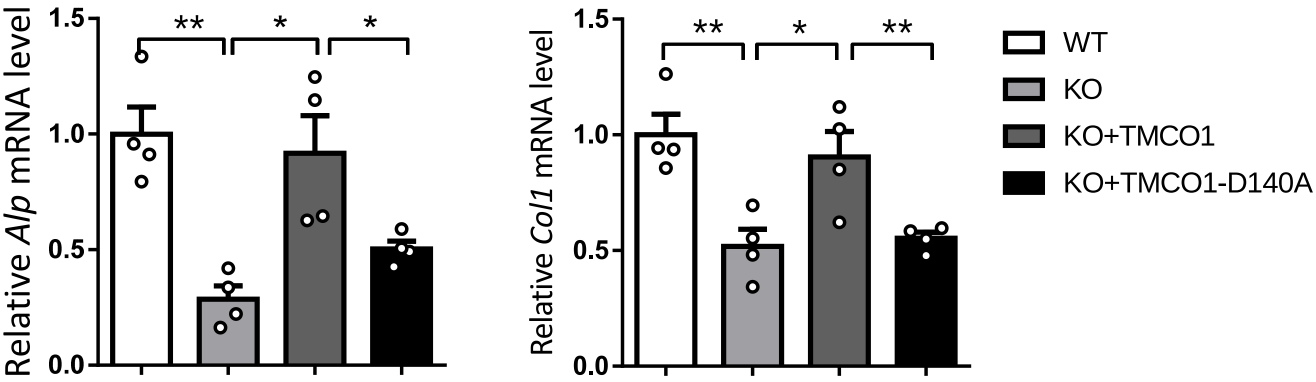


**Supplementary Figure 8. Effect of TMCO1 on osteoblast function is dependent on its calcium permeability.** The expression of osteoblast marker genes in WT and *Tmco1*$^{-/-}$ osteoblasts and *Tmco1*$^{-/-}$ osteoblasts expressing wild-type and mutated TMCO1. Representative results of three independent experiments are shown. Data are presented as the mean ± s.e.m. one-way ANOVA with multiple comparison test, *$P$ < 0.05 and **$P$ < 0.01.

## Supplementary Figure 9

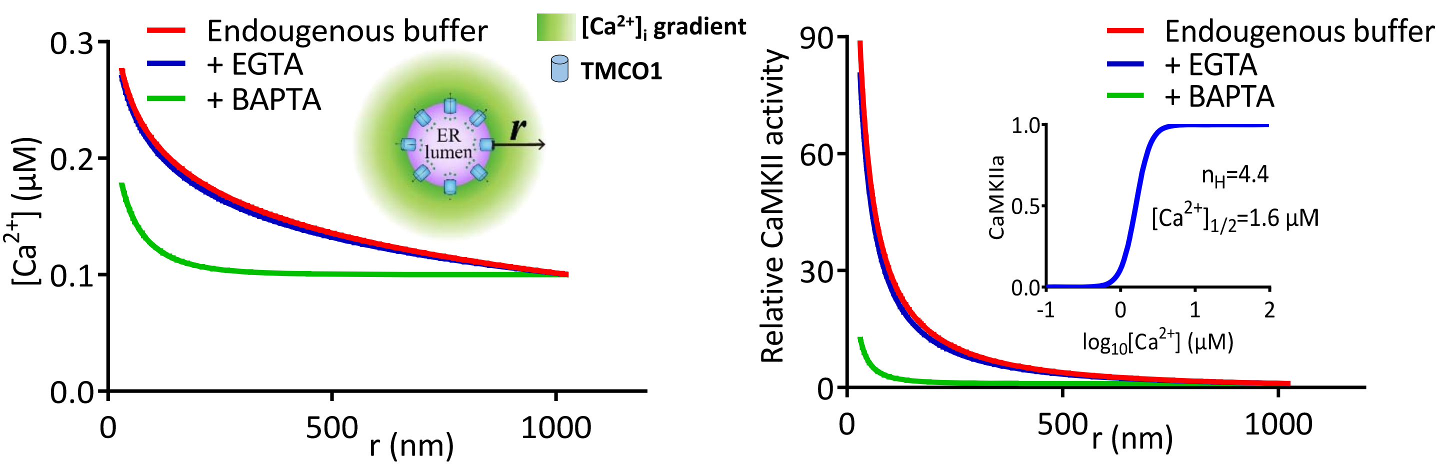


**Supplementary Figure 9. [$Ca^{2+}$] and CaMKII activity profiles near ER tubules.** In this simulation, [$Ca^{2+}$] accumulation and CaMKII activation stem from steady calcium leakage from channels uniformly distributed on the tubule surface. Calcium from ER tubules can freely diffuse, bind to the buffer and diffuse in the form of calcium buffer or dissociate from the buffer. [$Ca^{2+}$] (left) and CaMKII activity (right) relative to CaMKII activity in the resting state were simulated in three different cases: basal conditions with endogenous buffer (shown with red lines), EGTA addition (shown with blue lines) and BAPTA addition (shown with black lines). CaMKII activity was calculated based on the Hill equation and fitted to the calcium titration data in Bradshaw *et al*[39].

# Supplementary Figure 10

Uncropped blots for Figure 1a

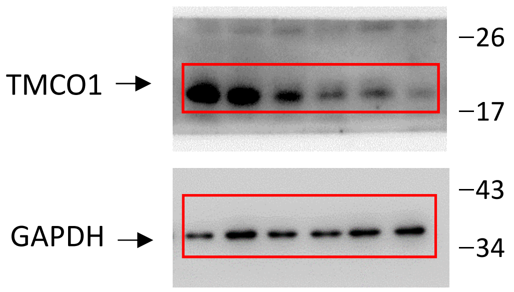


Uncropped blots for Figure 1c

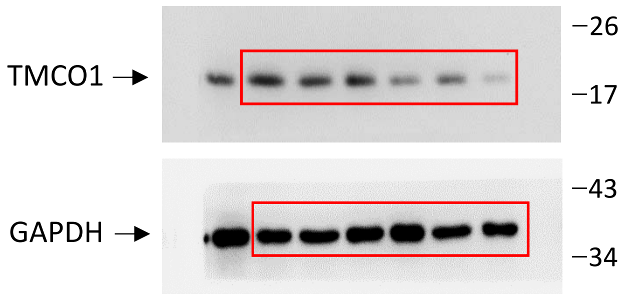


Uncropped blots for Figure 1d

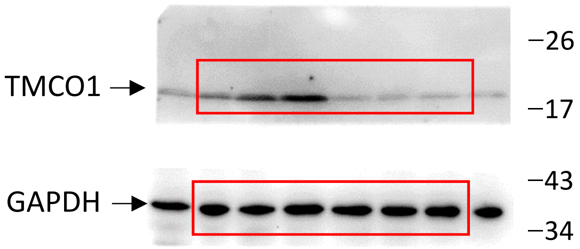


Uncropped blots for Figure 2e

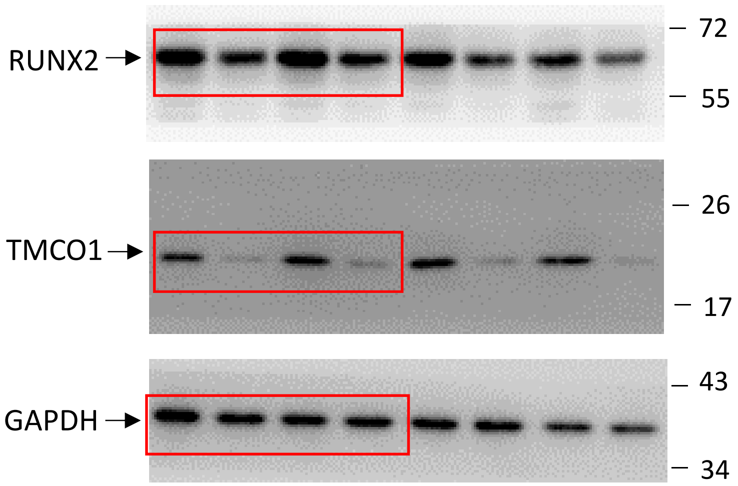


Uncropped blots for Figure 2f

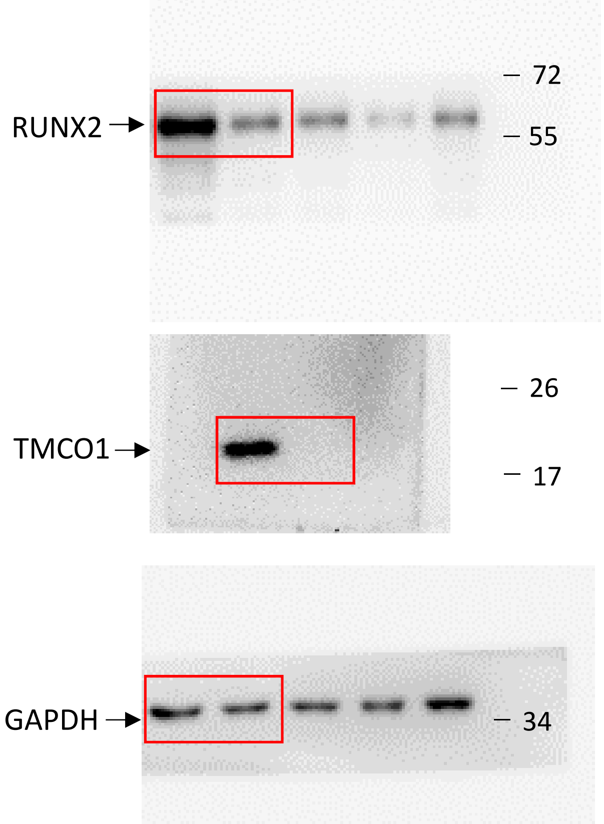


Uncropped blots for Figure 2g

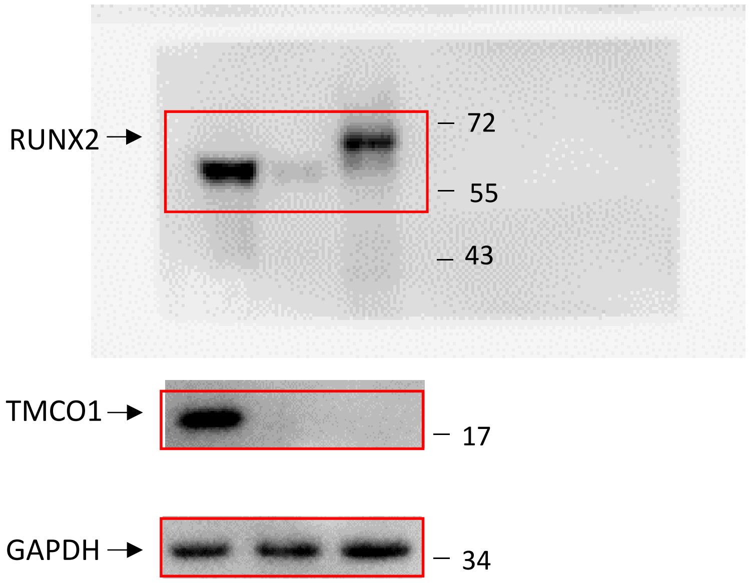


**Supplementary Figure 10. Uncropped western blots.** Red boxes in the uncropped blots indicate the cropped regions shown in the corresponding figures.

## Supplementary Figure 10 (continued)

Uncropped blots for Figure 3a

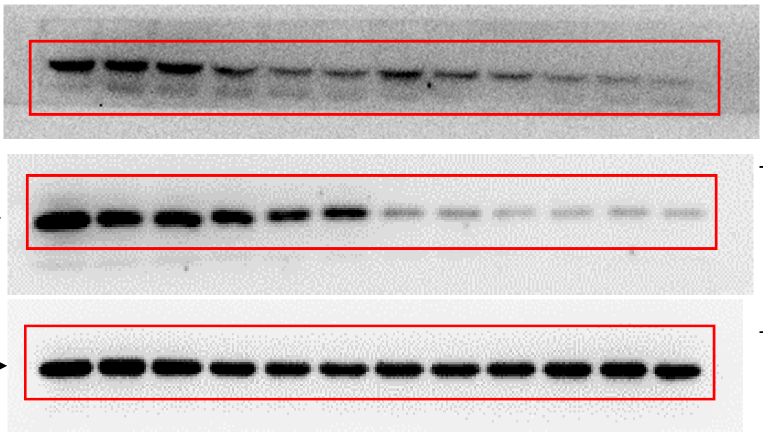


Uncropped blots for Figure 3b

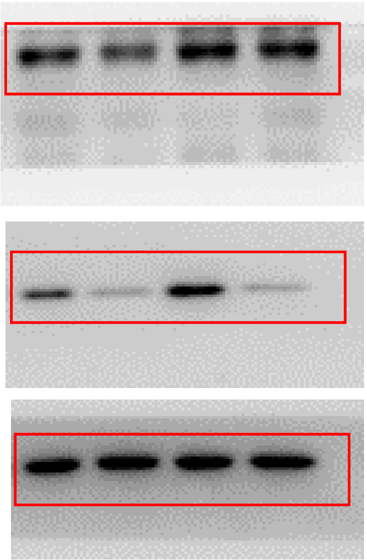


Uncropped blots for Figure 3c

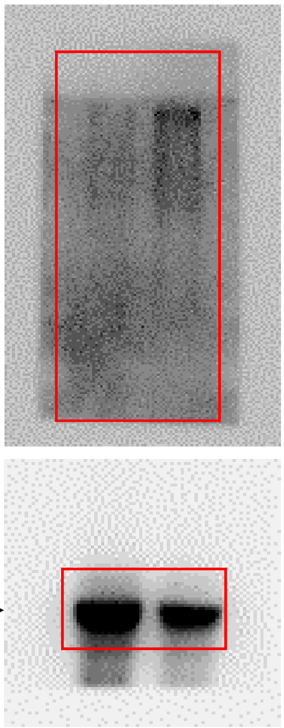


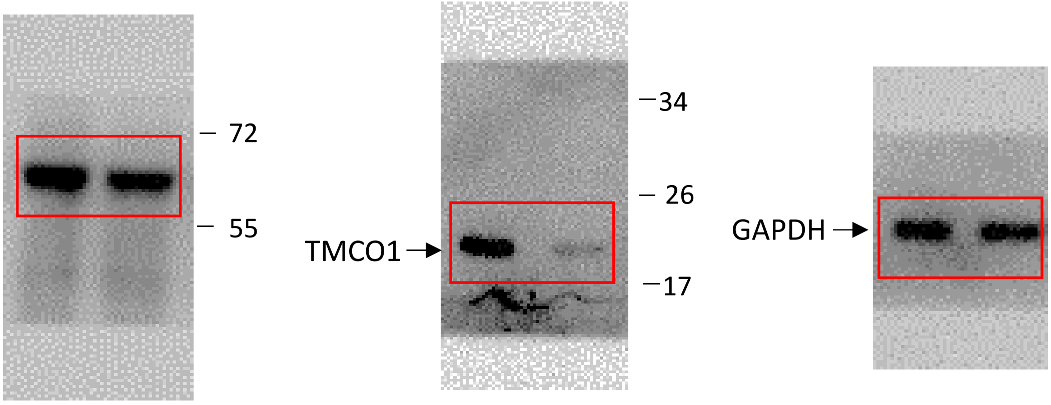

# Supplementary Figure 10 (continued)

Uncropped blots for Figure 3d

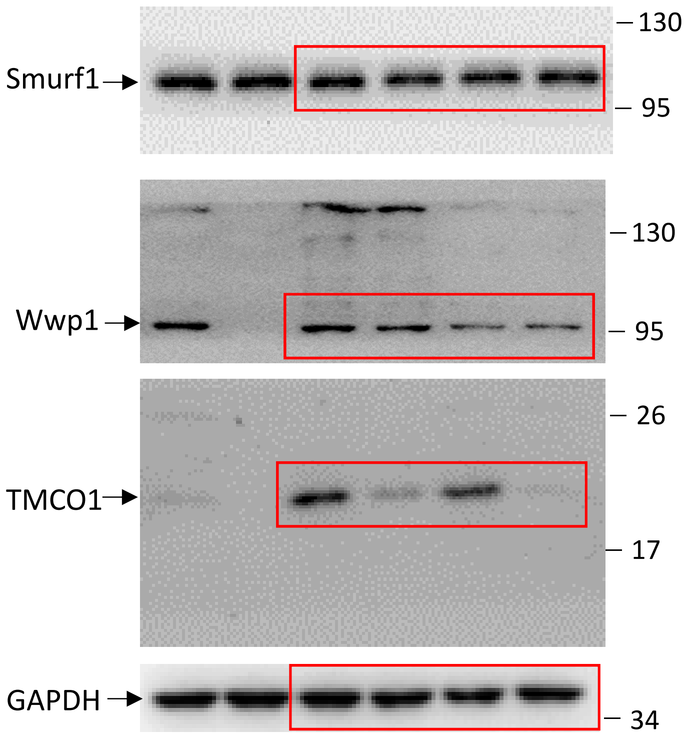


Uncropped blots for Figure 3e

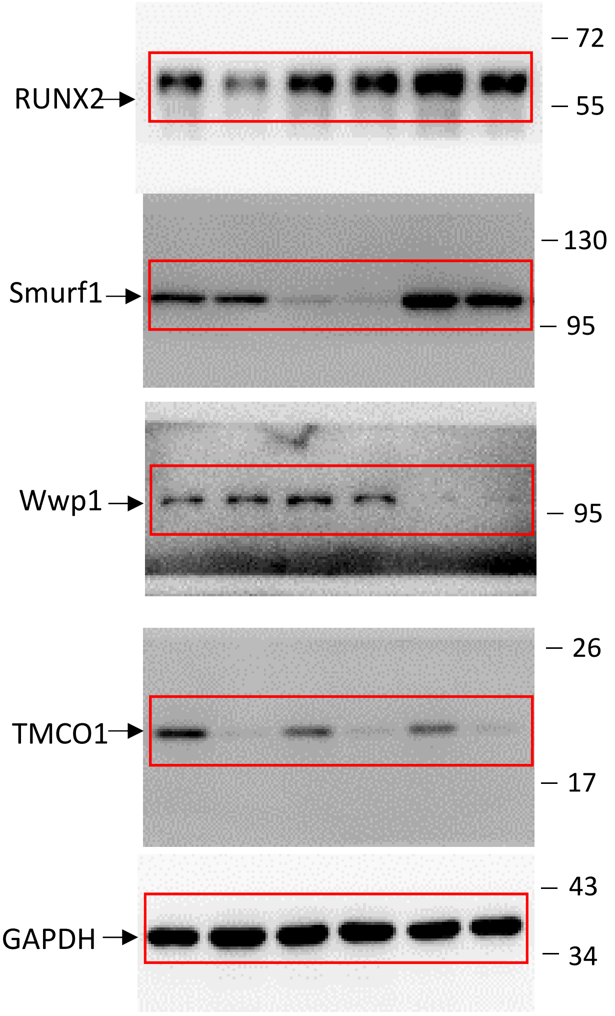


Uncropped blots for Figure 3f

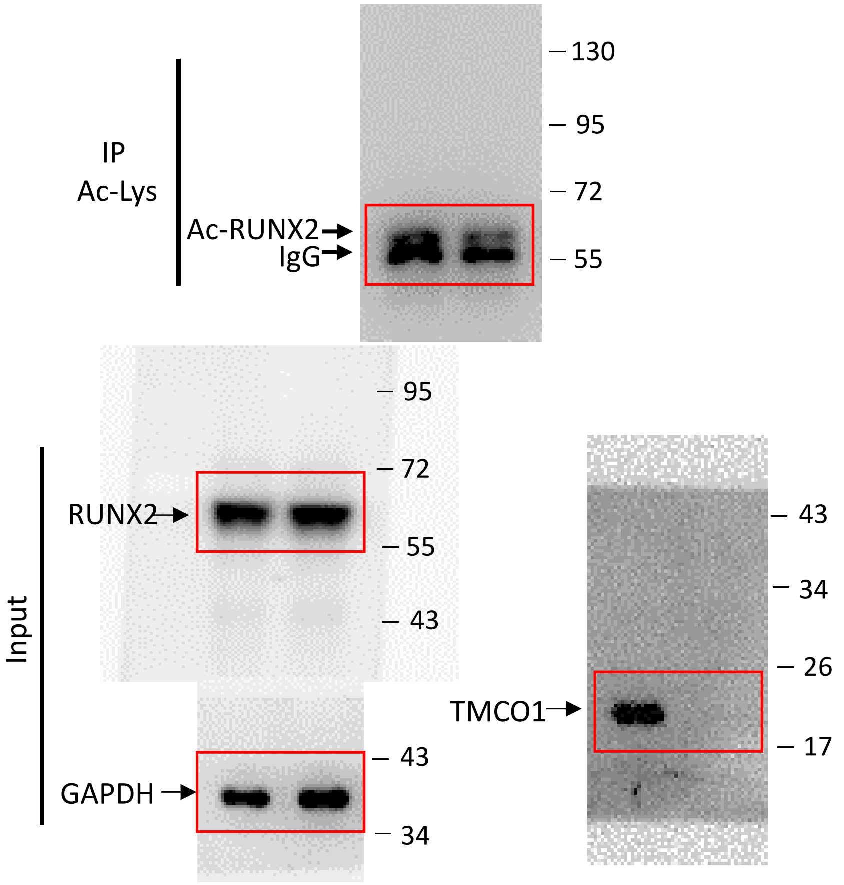


Uncropped blots for Figure 3g

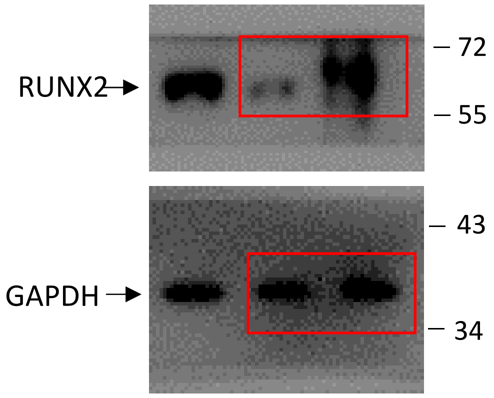

# Supplementary Figure 10 (continued)

Uncropped blots for Figure 4a

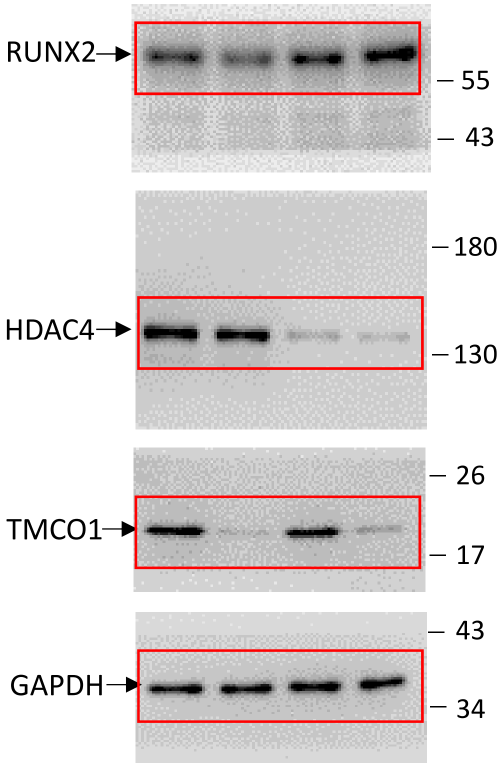


Uncropped blots for Figure 4c

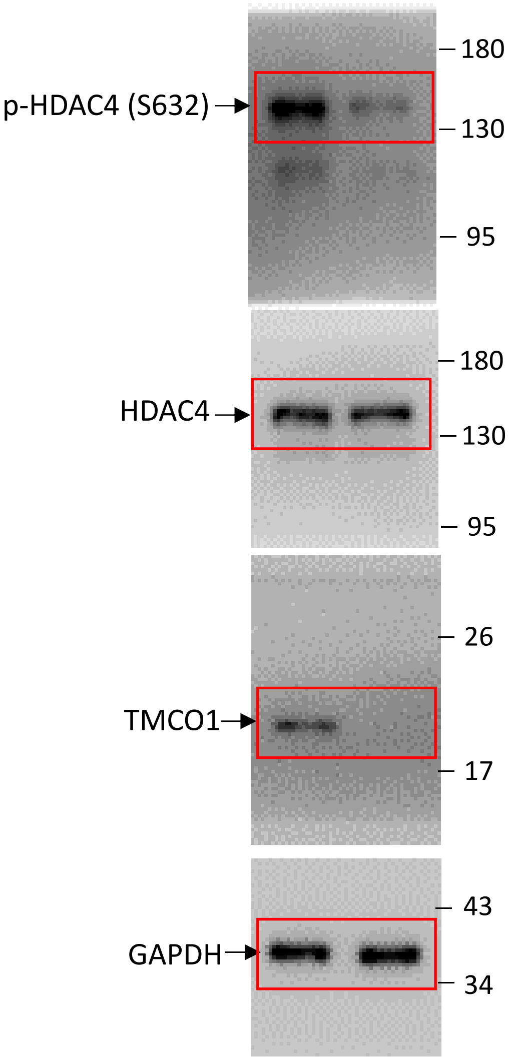


Uncropped blots for Figure 4d

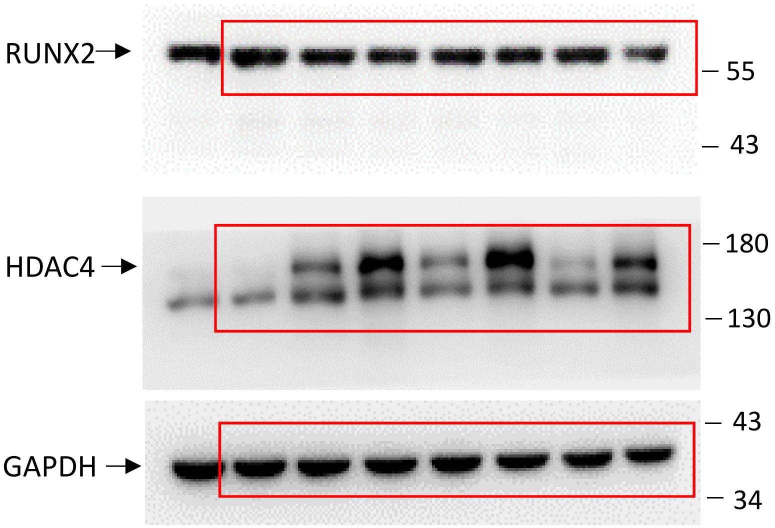

# Supplementary Figure 10 (continued)

Uncropped blots for Figure 5a

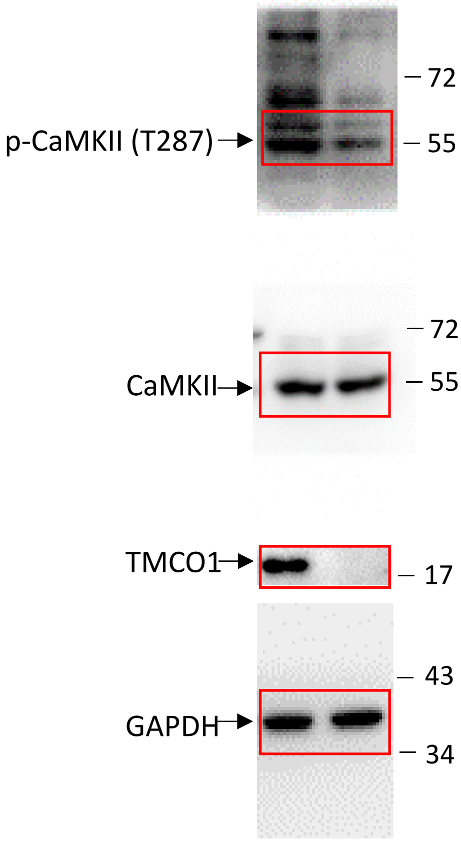


Uncropped blots for Figure 5b

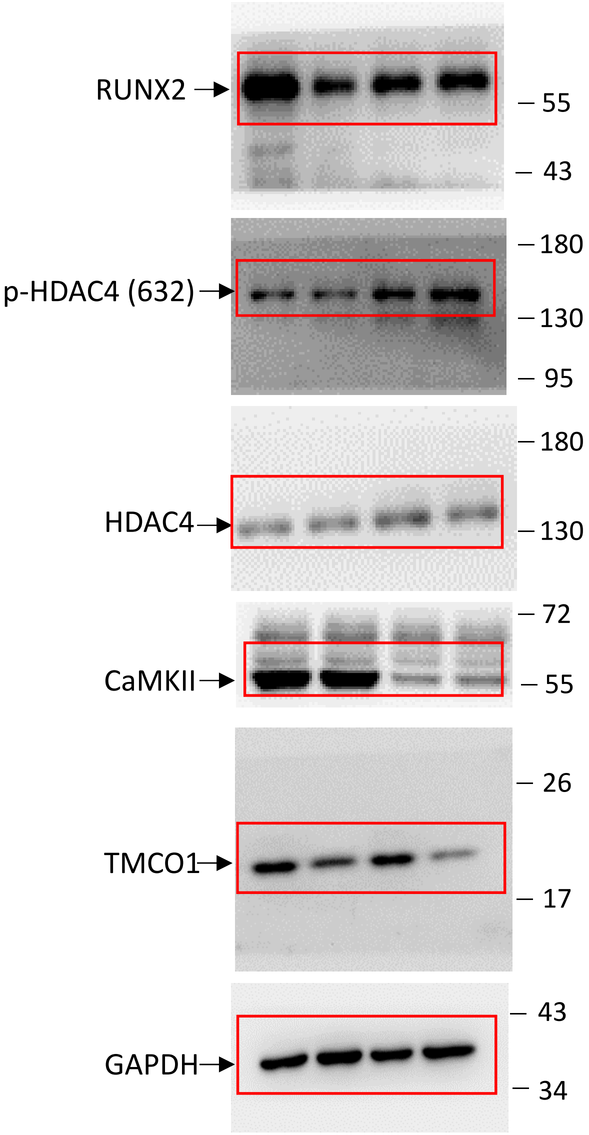


Uncropped blots for Figure 5c

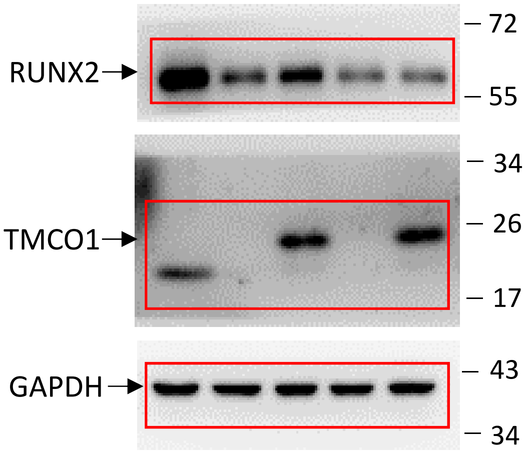


Uncropped blots for Figure 5d

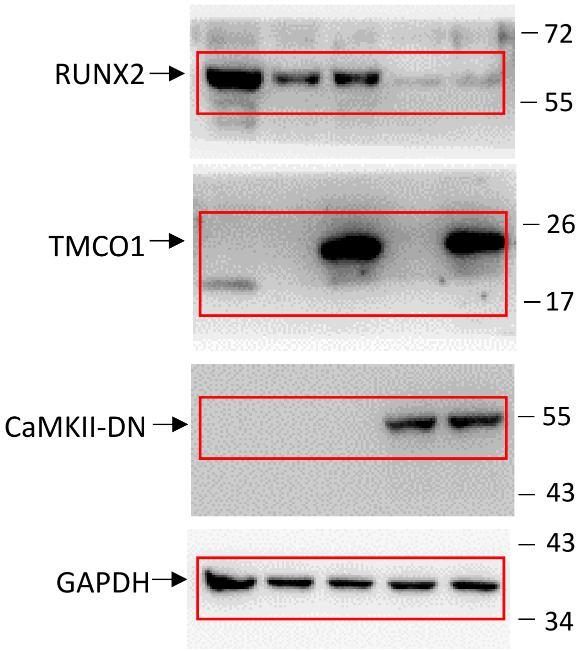

## Supplementary Figure 10 (continued)

Uncropped blots for Figure 6d

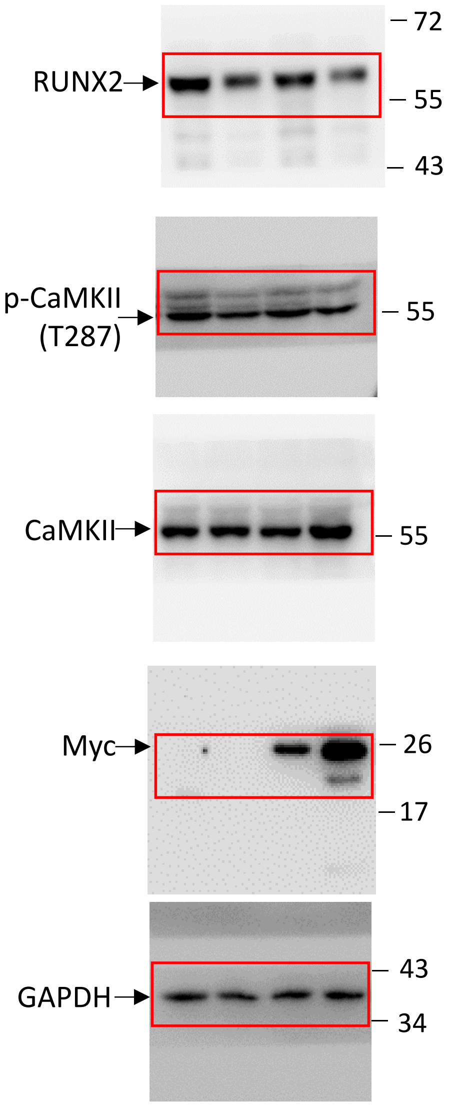


Uncropped blots for Figure 6e

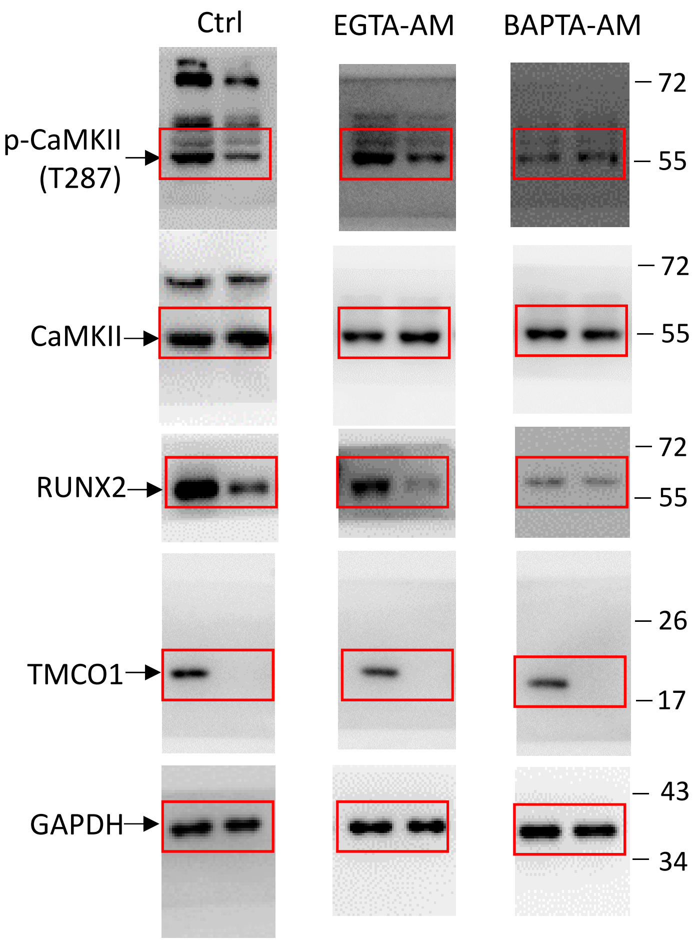

# Supplementary Figure 10 (continued)

Uncropped blots for Supplementary Figure 2b

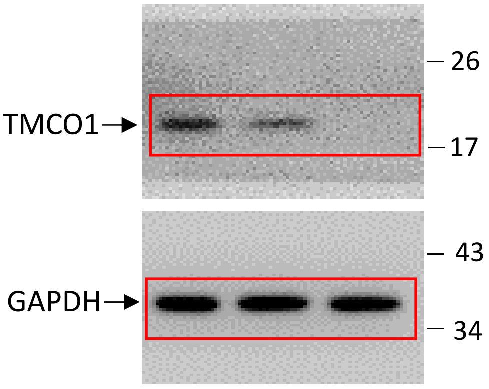


Uncropped blots for Supplementary Figure 3c

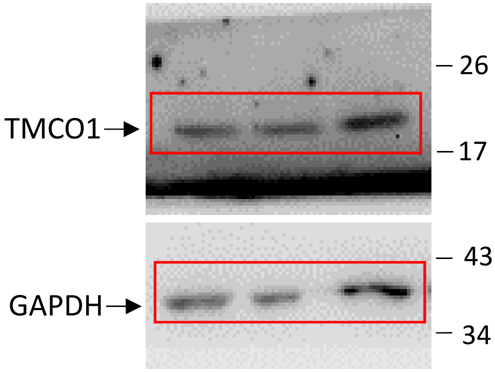


Uncropped blots for Supplementary Figure 4a

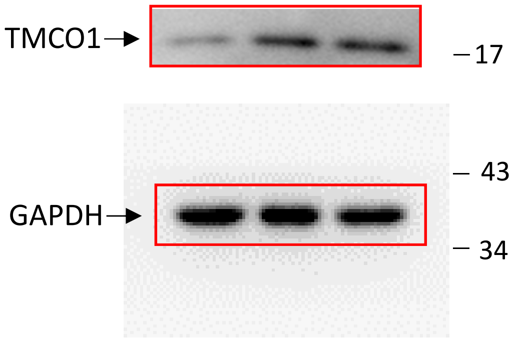


Uncropped blots for Supplementary Figure 4b

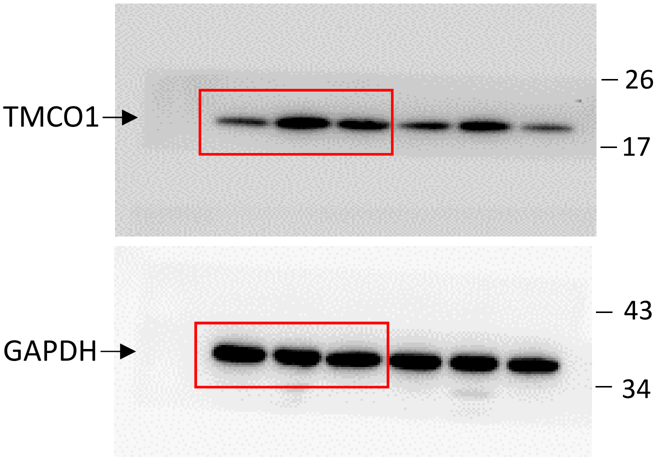


Uncropped blots for Supplementary Figure 5d

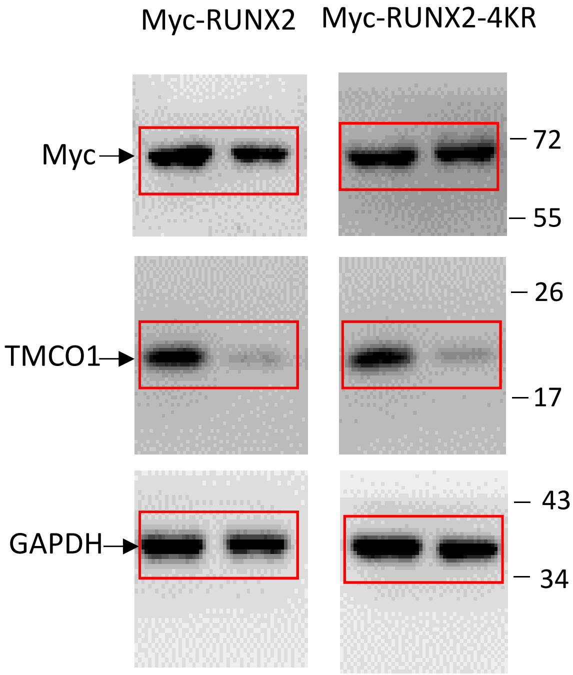


Uncropped blots for Supplementary Figure 6b

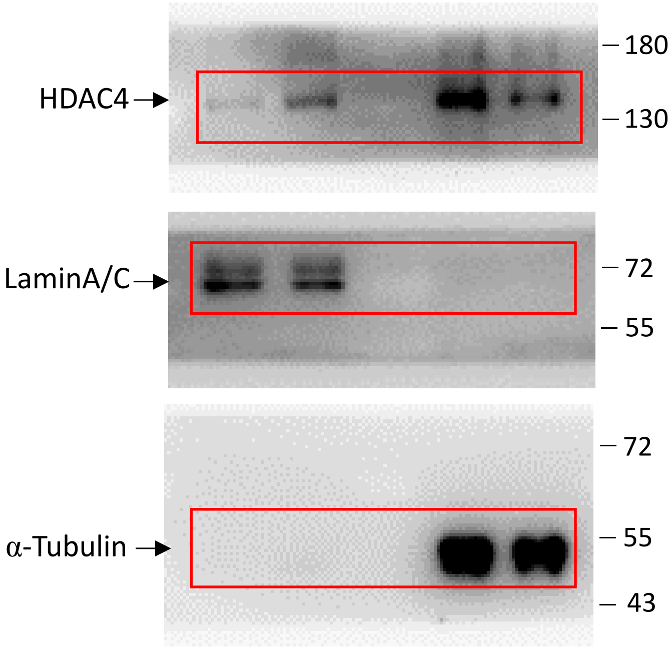

## Supplementary Table 1 Clinical features of fracture patients involved in bone specimens analysis

| Patient | **Age** | **Gender** | **T score for BMD at Lumbar Spine** | **Diagnosis** | **P1NP (ng/ml)** | **β-CTX (ng/ml)** |
|---|---|---|---|---|---|---|
| 1 | 81 | Female | 0.60 | Femoral Neck Fracture (Left) | 33.29 | 0.62 |
| 2 | 85 | Female | 0.20 | Femoral Neck Fracture (Left) | 26.07 | 0.23 |
| 3 | 73 | Female | -0.20 | Femoral Neck Fracture (Right) | 94.77 | 0.96 |
| 4 | 84 | Female | -0.50 | Femoral Neck Fracture (Left) | 168.4 | 0.97 |
| 5 | 74 | Female | -0.80 | Femoral Neck Fracture (Right) | 22.98 | 0.27 |
| 6 | 79 | Female | -1.3 | Femoral Neck Fracture (Left) | 73.58 | 0.92 |
| 7 | 83 | Female | -1.5 | Femoral Neck Fracture (Right) | 77.91 | 0.67 |
| 8 | 63 | Female | -1.7 | Femoral Neck Fracture (Left) | 43.74 | 0.28 |
| 9 | 68 | Female | -1.8 | Femoral Neck Fracture (Left) | 85.40 | 0.99 |
| 10 | 84 | Female | -2.0 | Femoral Neck Fracture (Right) | 45.81 | 0.86 |
| 11 | 93 | Female | -2.2 | Femoral Neck Fracture (Right) | 31.41 | 0.52 |
| 12 | 68 | Female | -2.5 | Femoral Neck Fracture (Left) | 28.65 | 0.92 |
| 13 | 74 | Female | -2.6 | Femoral Neck Fracture (Left) | 36.21 | 0.51 |
| 14 | 75 | Female | -2.7 | Femoral Neck Fracture (Left) | 47.82 | 0.97 |
| 15 | 71 | Female | -2.8 | Femoral Neck Fracture (Left) | 38.70 | 0.84 |
| 16 | 89 | Female | -2.9 | Femoral Neck Fracture (Right) | 32.95 | 0.62 |
| 17 | 73 | Female | -3.0 | Femoral Neck Fracture (Right) | 79.45 | 0.96 |
| 18 | 81 | Female | -4.1 | Femoral Neck Fracture (Right) | 63.13 | 0.55 |

| Patient | SF (ng/ml) | Hb (g/L) | Crea (mmol/L) | BUN (mmol/L) | Ca (mmol/L) | Ua (mmol/L) | ALB (g/L) | ALT (IU/L) | AST (IU/L) | GLU (mmol/L) |
|---|---|---|---|---|---|---|---|---|---|---|
| 1 | 188.0 | 127 | 61 | 4.74 | 2.15 | 231 | 44.9 | 38 | 29 | 5.88 |
| 2 | 142.0 | 112 | 78 | 5.62 | 2.12 | 301 | 42.3 | 14 | 23 | 6.17 |
| 3 | 296.5 | 120 | 72 | 3.63 | 2.29 | 293 | 46.6 | 15 | 18 | 5.39 |
| 4 | 196.4 | 107 | 62 | 2.03 | 2.13 | 143 | 33.1 | 11 | 17 | 6.56 |
| 5 | 483.7 | 118 | 47 | 4.65 | 2.10 | 158 | 41.1 | 16 | 17 | 5.74 |
| 6 | 192.6 | 140 | 64 | 7.21 | 2.11 | 291 | 44.0 | 27 | 23 | 6.34 |
| 7 | 267.0 | 121 | 91 | 7.91 | 2.34 | 232 | 39.6 | 10 | 18 | 6.24 |
| 8 | 306.1 | 125 | 65 | 6.94 | 2.33 | 298 | 41.6 | 8 | 19 | 7.37 |
| 9 | 92.00 | 93 | 77 | 3.79 | 2.12 | 202 | 38.5 | 12 | 17 | 4.47 |
| 10 | 685.2 | 97 | 114 | 8.75 | 2.15 | 438 | 37.5 | 10 | 14 | 6.33 |
| 11 | 299.0 | 130 | 54 | 5.58 | 2.16 | 174 | 32.4 | 18 | 22 | 6.88 |
| 12 | 262.2 | 119 | 54 | 6.81 | 2.20 | 384 | 39.0 | 13 | 14 | 5.09 |
| 13 | 705.6 | 121 | 39 | 3.83 | 2.08 | 110 | 29.9 | 11 | 15 | 6.92 |
| 14 | 416.7 | 118 | 87 | 9.27 | 2.14 | 348 | 40.1 | 20 | 23 | 4.91 |
| 15 | 399.5 | 146 | 65 | 5.27 | 2.37 | 249 | 49.0 | 14 | 20 | 6.96 |
| 16 | 169.0 | 113 | 68 | 5.72 | 2.18 | 180 | 27.6 | 8 | 17 | 5.46 |
| 17 | 98.00 | 108 | 57 | 4.78 | 2.03 | 313 | 39.0 | 23 | 26 | 5.84 |
| 18 | 298.2 | 112 | 86 | 6.43 | 2.17 | 247 | 37.4 | 17 | 19 | 5.40 |
