# Supplementary material for: TMCO1-mediated Ca2+ leak underlies osteoblast functions via CaMKII signaling
Source: Nat Commun. 2019 Apr 8;10:1589. doi: 10.1038/s41467-019-09653-5 (PMC6453895; doi:10.1038/s41467-019-09653-5)
Supplement: Supplementary file 2 — Reporting Summary [file 41467_2019_9653_MOESM2_ESM.pdf]

nature research

Corresponding author(s): Yingxian Li

Last updated by author(s): Feb 20, 2019

Reporting Summary

Nature Research wishes to improve the reproducibility of the work that we publish. This form provides structure for consistency and transparency in reporting. For further information on Nature Research policies, see Authors & Referees and the Editorial Policy Checklist.

Statistics

For all statistical analyses, confirm that the following items are present in the figure legend, table legend, main text, or Methods section.

n/a	Confirmed	
☐	☒	The exact sample size (*n*) for each experimental group/condition, given as a discrete number and unit of measurement
☐	☒	A statement on whether measurements were taken from distinct samples or whether the same sample was measured repeatedly
☐	☒	The statistical test(s) used AND whether they are one- or two-sided<br>*Only common tests should be described solely by name; describe more complex techniques in the Methods section.*
☐	☒	A description of all covariates tested
☐	☒	A description of any assumptions or corrections, such as tests of normality and adjustment for multiple comparisons
☐	☒	A full description of the statistical parameters including central tendency (e.g. means) or other basic estimates (e.g. regression coefficient) AND variation (e.g. standard deviation) or associated estimates of uncertainty (e.g. confidence intervals)
☐	☒	For null hypothesis testing, the test statistic (e.g. *F*, *t*, *r*) with confidence intervals, effect sizes, degrees of freedom and *P* value noted<br>*Give P values as exact values whenever suitable.*
☒	☐	For Bayesian analysis, information on the choice of priors and Markov chain Monte Carlo settings
☒	☐	For hierarchical and complex designs, identification of the appropriate level for tests and full reporting of outcomes
☒	☐	Estimates of effect sizes (e.g. Cohen's *d*, Pearson's *r*), indicating how they were calculated

Our web collection on statistics for biologists contains articles on many of the points above.

Software and code

Policy information about availability of computer code

Data collection	ZEN 2 (blue edition, ZEISS)
Data analysis	Interactive Data Language (IDL, Research Systems), GraphPad Prism 6.0

For manuscripts utilizing custom algorithms or software that are central to the research but not yet described in published literature, software must be made available to editors/reviewers. We strongly encourage code deposition in a community repository (e.g. GitHub). See the Nature Research guidelines for submitting code & software for further information.

Data

Policy information about availability of data

All manuscripts must include a data availability statement. This statement should provide the following information, where applicable:

- Accession codes, unique identifiers, or web links for publicly available datasets
- A list of figures that have associated raw data
- A description of any restrictions on data availability

The figure source data were provided in the supplementary information file.

Field-specific reporting

Please select the one below that is the best fit for your research. If you are not sure, read the appropriate sections before making your selection.

☒ Life sciences ☐ Behavioural & social sciences ☐ Ecological, evolutionary & environmental sciences

For a reference copy of the document with all sections, see nature.com/documents/nr-reporting-summary-flat.pdf

Life sciences study design

All studies must disclose on these points even when the disclosure is negative.

Sample size	No statistical method was used to predetermine sample size. We chose the numbers of mice to study based on our prior experience that give good standard errors of the mean and good statistics to make it unlikely that we miss a biologically important difference between groups.
Data exclusions	Some of the data were excluded from the study because of the pre-established criteria such as problems or failures in identifying correct genotypes or birth dates, and issues unrelated to the intervention of the study such as spontaneous malnutrition. In any case, we consistently used littermate controls with corresponding genotypes in analysis.
Replication	For all data presented in the manuscript, we examined at least three independent biological samples (three different mice) to ensure the reproducibility. For each series of the experiments, all attempts at replication were successful.
Randomization	Animals and cells were randomly assigned to the experimental groups.
Blinding	Investigators were blinded to groups allocation during data collection.

Reporting for specific materials, systems and methods

We require information from authors about some types of materials, experimental systems and methods used in many studies. Here, indicate whether each material, system or method listed is relevant to your study. If you are not sure if a list item applies to your research, read the appropriate section before selecting a response.

Materials & experimental systems

n/a	Involved in the study
☐	☒ Antibodies
☐	☒ Eukaryotic cell lines
☒	☐ Palaeontology
☐	☒ Animals and other organisms
☐	☒ Human research participants
☒	☐ Clinical data

Methods

n/a	Involved in the study
☒	☐ ChIP-seq
☒	☐ Flow cytometry
☒	☐ MRI-based neuroimaging

Antibodies

Antibodies used	rabbit anti-TMCO1 (1:500, Sigma, CatNo. AV49429, polyclonal, LotNo. 023MF1473B), rabbit anti-Wwp1 (1:500, Sigma, CatNo. SAB2102717, polyclonal, LotNo. 11132-1A7), rabbit anti-RUNX2 (1:1000, Cell Signaling Technology, CatNo. 8486, D1H7, LotNo. 2), mouse anti-HDAC4 (1:1000, Cell Signaling Technology, CatNo. 5392, 4A3, LotNo. 1), rabbit anti-p-HDAC4 (1:1000, S632, Cell Signaling Technology, CatNo. 3424, polyclonal, LotNo. 3), mouse anti-Myc-Tag (1:1000, Cell Signaling Technology, CatNo. 2276, 9B11, LotNo. 24), rabbit anti-CaMKII (1:1000, GeneTex, CatNo. GTX111401, polyclonal, LotNo. 42018), rabbit anti-p-CaMKII (1:1000, T287, GeneTex, CatNo. GTX52342, polyclonal, LotNo. 821704329), rabbit anti-Smurf1 (1:1000, Abcam, CatNo. ab38866, polyclonal), GAPDH (1:5000, Abclonal Technology, CatNo. AC033, LotNo. 9100033001), rabbit anti-Ac-K2-100 (1:100, Cell Signaling Technology, CatNo. 9814, mAb mix, LotNo. 5), mouse anti-ubiquitin antibody (1:1000, Cell Signaling Technology, CatNo. 3936, P4D1, LotNo. 13), rabbit anti-HDAC4 (1:100, Proteintech, CatNo. 17449-1-AP, polyclonal, LotNo. 00018711), rabbit anti-osteocalcin (1:100, Proteintech, CatNo. 23418-1-AP, polyclonal, LotNo.00045673) and mouse anti-Col1a (1:400, Abcam, CatNo. ab96723, polyclonal, LotNo. GR247379-39).
Validation	More detailed information about these antibodies is available on these manufacturers' websites.

Eukaryotic cell lines

Policy information about cell lines

Cell line source(s)	HEK293A, C3H10T1/2, MC3T3-E1 cells from National Infrastructure of Cell Line Resource (Beijing, China)
Authentication	All of the cell lines were purchased from National Infrastructure of Cell Line Resource (Beijing, China).
Mycoplasma contamination	All cell lines were routinely tested for mycoplasma contamination.
Commonly misidentified lines (See ICLAC register)	No cell line listed by ICLAC was used.

Animals and other organisms

Policy information about studies involving animals; ARRIVE guidelines recommended for reporting animal research

Laboratory animals	We used genetically modified mice (mus musculus) for this study. Most of the mouse lines have been backcrossed to a C57/BL6 background. Male mice were used throughout this project.
Wild animals	The study did not involve wild animals.
Field-collected samples	The study did not involve samples collected from the field.
Ethics oversight	All the experimental procedures were approved by the Committees of Animal Ethics and Experimental Safety of China Astronaut Research and Training Center.

Note that full information on the approval of the study protocol must also be provided in the manuscript.

Human research participants

Policy information about studies involving human research participants

Population characteristics	Patients who had fracture caused by falling without obvious violence from the Second Affiliated Hospital of Soochow University from January 2016 to January 2017 were included in our study (inclusive criteria). Excluding criteria as below: (1) Participants with fracture caused by high-energy injury and pathologic fracture due to other diseases were excluded; (2) participants with hyperparathyroidism, other congenital or acquired bone disease, history of malignancy, significant liver, or renal disease; (3) participants that had taken glucocorticoids or estrogen, or selective estrogen receptor modulators within 6 months; (4) participants that had ever taken parenteral bisphosphonates, teriparatide, calcitonin, or strontium ranelate within 12 months. Above all, we recruited 18 female patients aged 60 years or older with fracture caused by low-energy injury.
Recruitment	We recruited 18 female patients aged 60 years or older with fracture caused by low-energy injury. The osteoporosis group was defined as a BMD T score of ≤−2.5 at the lumbar, and a T score of ＞－2.5 as the normal group. We obtained informed consent from the participants.
Ethics oversight	All the clinical procedures were approved by the Committees of Clinical Ethics in the Second Affiliated Hospital of Soochow University (Suzhou, China).

Note that full information on the approval of the study protocol must also be provided in the manuscript.
